# Durability of Flame-Retarded, Co-Extruded Profiles Based on High-Density Polyethylene and Wheat Straw Residues

**DOI:** 10.3390/molecules26113217

**Published:** 2021-05-27

**Authors:** Arne Schirp, Jan Dannenberg

**Affiliations:** 1 Fraunhofer-Institute for Wood Research (WKI), 38108 Braunschweig, Germany; 2 Lowke Schiessl Ingenieure GmbH, 38106 Braunschweig, Germany; dannenberg@lowke-ing.de

**Keywords:** fire retardancy, weathering, durability, cone calorimetry, co-extrusion, polyethylene, thermoplastic, wheat straw, composites

## Abstract

At present, little information is available in the scientific literature related to the durability (weathering resistance) of fire-retarded wood and natural fiber-reinforced thermoplastics. In this work, thermoplastic profiles for façade applications based on high-density polyethylene, wheat straw particles, and fire-retardants were extruded and their reaction-to-fire performance before and after artificial weathering evaluated. Profile geometries were either solid or hollow-core profiles, and fire-retardants (FR) were added either in the co-extruded layer or in the bulk. Various FR for inclusion in the co-extruded layer were screened based on UL-94 tests. For profile extrusion, two types of FR were chosen: a coated intumescent combination based on ammonium polyphosphate (APP) and an APP coated with melamine and without formaldehyde. Before weathering, the peak heat release rate (pHRR) and the total heat release (THR), which were determined using cone calorimeter measurements, were reduced by up to 64% and 67% due to the FR. However, even before weathering, pHRR of the profiles was relatively high, with best (lowest) values between 230 and 250 kW/m^2^ under the test conditions. After 28 days of artificial weathering, changes in reaction-to-fire performance and color were evaluated. Use of the APP in the co-extruded layer worsened color change compared to the formulation without APP but the pHRR was not significantly changed. The influence of weathering on the fire behavior was small compared to the difference between fire-retarded and non-fire-retarded materials. Results from the cone calorimeter were analyzed with regard to ETAG 028, which provides requirements related to the durability of fire performance of building products. In many formulations, increase in THR was less than 20% compared to before weathering, which would place some of the profiles in class C or better (EN 13501-1). However, due to the high pHRR, at best, class D was obtained under the conditions of this study. In addition to cone calorimeter measurements, results from the single flame source test, limiting oxygen index determination and thermogravimetric analysis, are shown and discussed. Strength properties, water uptake and swelling of the profiles, thermal conductivity, and energy dispersive X-ray data are also presented.

## 1. Introduction

Natural fibers, wood particles, and agricultural residues represent a sustainable resource for a wide variety of composite materials. One of the most abundant agricultural residues is wheat straw, with an estimated annual production of 1000 million tons [1]. Traditionally, wheat straw has been used for low-value purposes, such as animal feeding, mulch, and bedding materials for animals. Often, straw is burnt on agricultural fields, although legislation has been enacted in many countries to prohibit this routine. It is desirable to create more high-value applications for straw residues. One option that has been explored is the manufacturing of straw particleboards bonded with methylene diphenyl diisocyanate resins. Wheat straw has also been widely used as filler in thermoplastic [2,3,4,5] and thermoset [6] composites.

Due to the relatively high amount of silica, wheat straw is interesting as filler to increase the fire-retardancy of composites. Wheat straw has a silica content of 3–9%, while ash content amounts to 8–13% [7]. Usually, a low amount of ash is equivalent to low silica content in annual plants. However, this relationship is not applicable for all annual plants, for example, rape straw. Other straw types such as rice straw have also attracted interest as low-cost filler. Dahy [8] investigated the flammability of polylactic acid (PLA)-lignin-rice straw composites prepared by thermoforming. Due to their high silica content, straw fibers were regarded as partial replacement for traditional fire-retardants (FR).

Fire-retardants such as ammonium polyphosphate (APP), expandable graphite (EG), aluminum and magnesium hydroxide, and zinc borate, which have been used in the production of wood-plastic composites (WPC), are also applicable for processing straw-plastic composites. Usually, FR are added during the compounding step prior to profile extrusion but a cost-efficient option is to add the FR in a co-extruded layer and to omit the FR in the bulk of the profile [9].

In addition to profile extrusion, injection-molding has also been used to process fire-retarded, halogen-free WPC formulations [10]. The authors investigated various combinations of FR in a polypropylene (PP) matrix with wood particles as filler and observed the best overall results with mixtures based on 15 wt.% EG or EG/APP combinations at different ratios. The combination of EG and APP modified with 3-(methylacryloxyl) propyltrimethoxy silane as FR system in PP-based WPC was investigated by Guo et al. [11]. Another option is to improve the fire retardancy of WPC is the addition of biochar [12], which has the benefit of enhancing certain mechanical properties, such as flexural strength and modulus. Flammability and fire resistance of composites reinforced by wood and natural fibers has been reviewed by Kozlowski and Wladyka-Przybylak [13], Nikolaeva and Kärki [14], and Mngomezulu et al. [15].

A recent review by Sonnier et al. [16] covers the flame retardancy of natural fiber-reinforced composites with a focus on the differences in flammability between composites filled with natural fibers or glass and carbon fibers. Only few publications deal with the effects of weathering (artificial, natural) on the performance of fire-retarded, extruded profiles. Garcia et al. [17] performed accelerated weathering trials with fire-retarded WPC and focused on determining color changes due to FR with different light stabilizers. The reaction-to-fire performance after weathering was not investigated. Turku and Kärki [18] investigated the durability of fire-retarded wood-PP composites exposed to freeze-thaw cycling. They used five FR, namely, melamine, aluminum trihydrate, graphite, zinc borate, and titanium oxide in the co-extruded layer of PP-based profiles. Based on FT-IR spectroscopy measurements, it was shown that melamine and zinc borate leached partly from the surface during weathering. However, the effects of the leaching on fire performance were not determined in their paper. Nikolaeva and Kärki [9] used the cone calorimeter to evaluate the reaction-to-fire performance of co-extruded wood-PP composites. The performance after weathering was not addressed. Various FR were used in the shell layers in combination with pulp cellulose of unknown fiber size, whereas softwood fiber was used in the core layer.

Profile extrusion is one way to produce thermoplastic composites with straw fillers, with potential applications in the building and infrastructure sectors, such as for sidings, fences, and railings. To enable the application of siding profiles for building applications, the reaction-to fire performance has to be tested and information regarding the durability of the fire retardancy needs to be obtained. Most producers of siding profiles are interested in obtaining CE marking of their products, and to achieve this, reaction-to-fire testing is mandatory.

The European classification system (EN 13501-1, 2002) includes one system for all construction products except floorings and one system specifically for floorings. Most wood products fall into class D-s2; d0; or, for floorings, class Dfl-s1 [19]. The durability of fire-retardant treated wood products has been reviewed in the literature [20], but WPC have not been included so far. The durability of reaction-to-fire performance (classes of fire-retardant treated wood products in interior and exterior end use applications) has only recently been addressed in an international standard (EN 16755, 2017). EN 16755 applies to wood products that have been treated during a production process with fire retardant products applied either by a penetration process or by a superficial process, for example, an intumescent fire-retardant coating. The fire performance of a coated or, as in this research project, co-extruded profile can be investigated according to ISO 5660-1 (cone calorimeter) on a bench scale or according to EN 13823 (single-burning item (SBI) test) for actual products. Since testing according to EN 13823 requires a significant amount of material, in the present project, the cone calorimeter was used to obtain data pertaining to the reaction-to-fire performance of co-extruded profiles before and after artificial weathering.

In summary, it was the objective of this research project to determine the durability of co-extruded, fire-retarded wheat-straw-HDPE-composites for facades before and after artificial weathering. To evaluate the results of the weathering tests, ETAG 028 (2012) was applied. In addition, changes in mechanical and physical properties and color of the profiles were determined based on methods and requirements listed in the relevant standards EN 15534 parts 1 and 5. Thermogravimetric analysis (TGA) and SEM-EDX measurements were also performed with the fire-retarded composites.

## 2. Results and Discussion

### 2.1. Processing of Wheat Straw and Development of Fire-Retarded Co-Extruded Layers

**Figure 1:**
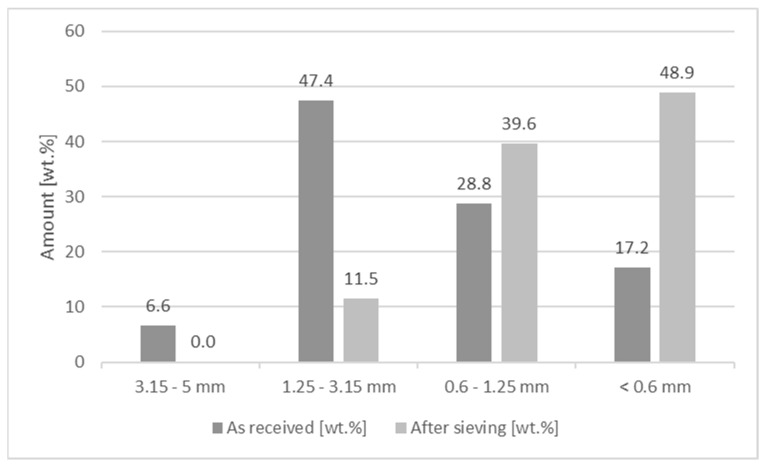
Processing of wheat straw particles for core and co-extruded layers: particle size distribution of particles before and after sieving in tumbler screening machine. Particles as received were used for the core, and particles after sieving in Condux mill with 1 mm sieve insert were used for the co-extruded layers.

**Figure 2:**
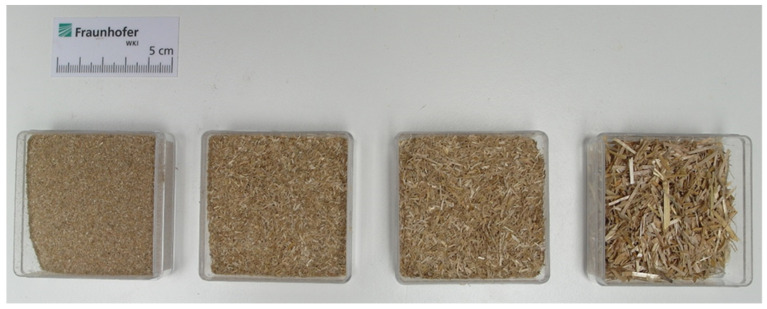
Particle size fractions after sieving. From left to right: 0.6–1.25 mm; 1.25–3.15 mm; 3.15–5 mm; >5 mm. Fraction < 0.6 mm not shown.

Particle size fractions of the wheat straw after sieving are shown in Figure 1. Particle size as received was mostly between 1.25 mm and 3.15 mm (47 wt.%), followed by the fractions: 0.6 mm to 1.25 mm (29 wt.%), smaller than 0.6 mm (17 wt.%), and 3.15 mm to 5 mm (7 wt.%). Since the target thickness of the co-extruded layer was 1 mm, only the fraction smaller than 0.6 mm was suitable but the yield was too low. Hence, the material was sieved in a Condux mill with 1 mm sieve insert. Of the resulting fractions, 49% of the particles were now smaller than 0.6 mm and could be used in the co-ex layer. Particles after sieving are shown in Figure 2.

The 0.6 mm particles were used for compounding with HDPE and FR, and the samples in sheet form were tested in the UL-94 vertical test (Table 1). With magnesium hydroxide (magnesium dihydroxide, MDH) as FR, the test was not passed, even when 50% of the FR were used. Magnesium hydroxide and aluminum trihydroxide (ATH) are the two most commonly used mineral flame retardants. The advantage of MDH is its higher decomposition temperature (approx. 340 °C) compared to ATH (180–200 °C). Metal hydroxides such as magnesium hydroxide have a requirement for relatively high loadings, but they are available at lower prices compared to other FR [21]. During combustion, metal hydroxides remove heat by releasing large amounts of water at the same temperature or below the temperature at which the decomposition of the polymer occurs. Hence, by absorbing heat, they slow down the polymer pyrolysis process, and the generated water vapors dilute the combustible gases of polymer decomposition. In addition, a non-flammable layer on the surface of the material is generated that protects the underlying substrate. A well-known advantage of metal hydroxides is that they contribute to smoke suppression.

Under the conditions of the present study, despite the high loading level, it appears that magnesium hydroxide is not effective as FR for the thin sheets based on HDPE and wheat straw, which mimic the co-extruded layer. In contrast, all other formulations except two reached V0-classification at 0.8 mm thickness and 30–40% loading levels in the UL-94 tests. All of the V0-classified formulations were based on APP. While magnesium hydroxide predominantly functions in the gas phase, APP works primarily in the condensed phase via char formation enhancement [22]. Usually, APP is combined with pentaerithrytol (PER) and melamine to obtain an intumescent system. A recommended ratio is often 3:1:1 for APP:PER:melamine. Intumescent flame retardants interrupt the self-sustained combustion of the polymer at its earliest stage [23]. Generally, intumescent systems consist of an acid source, e.g., ammonium polyphosphate or melamine polyphosphate; a carbonization agent, e.g., pentaerythrithol or, in this case, a lignocellulose such as wheat straw; and a blowing agent, e.g., melamine or urea. The development of an intumescence reaction and char formation are the result of the interaction of these three components. The acid source decomposes to a mineral acid that dehydrates the polyol to generate the carbon char, mainly by a free radical process. The carbon char is foamed by non-flammable gases emitted during the degradation of the blowing agent. Based on previous results [24] and in our experience, the use of PER is not required as additional carbonization agent due to the presence of a lignocellulose, in this case, wheat straw. However, there has been one report in which results from cone calorimetry indicated that the inclusion of PER in wood flour-PP-composites with APP may be beneficial [25]. The use of PER promoted the formation of honeycomb-like intumescent char and had a positive effect on the condensed-phase flame retardant mechanism. On the other hand, the addition of PER decreased the mechanical performance of the flame-retarded composites. It has been suggested that PER could interfere with the coupling agent [26], in this case, MAPE. This could not only lead to a loss in mechanical performance but also to increased water uptake and swelling of the composites.

In the present study, it was found that the different types of APP from different suppliers (Exolit AP 422 and Exolit AP 462 from Clariant; Budit 669 and FR CROS 490 from Budenheim) all worked equally well in UL94@0.8 mm when used in sufficiently high loading levels of 30–40%. Exolit AP 422 (Clariant) is uncoated, whereas Exolit AP 462 is microencapsulated with melamine. One of the products from Budenheim is a coated intumescent combination based on APP (Budit 669), whereas the other product is an APP phase II, coated with melamine, formaldehyde-free (FR CROS 490, Budenheim). APP phase or type II is of higher molecular weight, shows low solubility, and is only slowly hydrolyzable at ambient temperature [21]. It has also been reported that APP form II has partially crosslinked structure. The coating confers better water resistance by means of a durable thermoset aminoplast resin. Surface coatings based on melamine and aminoplast resins can also provide some intumescence to support flame retardancy.

The two APP from Budenheim were also tested at relatively low levels of 20%, with one product reaching V0-classification (FR CROS 490) and one product not passing under these conditions (Budit 669). Based purely on UL-94 tests, it was not possible to determine differences regarding the various APP types and to evaluate possible benefits of the synergists tested, i.e., expandable graphite (EG) and red phosphorous (RP). Expandable graphite can expand very quickly to over 100 times its original volume upon fast heating, as in the burning of a plastic matrix, hence producing a heat and mass transfer barrier. Such a barrier is composed of tiny worm-like fibrils, which are produced from individual graphite particles [21]. This effect is generated in almost any thermoplastic, and it is effective in polyolefins when combined with other FR such as APP, magnesium hydroxide or RP. The flame-retardant mechanism has been explained as a result of the formation of intumescent charred layers in the condensed phase that slow down heat and mass transfer between the gas and the condensed phases and limit the diffusion of oxygen to the polymer bulk [27]. A benefit of using EG in combination with APP or other FR is that formation of carbonaceous char is promoted and thermal stability of the materials is enhanced. The combination of EG and an intumescent system consisting of APP and a novel triazine-based char forming agent was used in wood flour-polypropylene composites by Bai et al. [28]. The authors report a synergistic effect between EG and the intumescent system (LOI value of 38.8% and UL-94 V-0 rating at 3 mm thickness) with 2:3-ratio of EG/intumescent system and 25 wt.% addition. In the present project, the combination of Exolit AP 422 and EG (20% APP and 10% EG) lead to V1-classification (in this case, at 0.8 mm thickness).

Seefeldt et al. [29] studied the effect of combinations of APP, EG, and RP for use in PP-based WPC in detail by using the cone calorimeter. Although EG showed the highest potential in its flame-retardant effect, expansion of EG led to disruption of the residue formed, thereby destroying the thermal barrier. By combining EG with APP and RP, the residue that was obtained from wood could be cross-linked.

Red phosphorus is flammable as a powder but available as a masterbatch, for example, in a low-density polyethylene (LDPE) matrix. It is a powerful flame-retardant additive and thermally stable up to ca. 450 °C [30]. Red phosphorus is particularly useful in glass-filled polyamide 6.6 where high processing temperatures (about 280 °C) exclude the use of most phosphorus compounds. Coated RP is often used to flame-retard polyamide-based electrical parts. In this study, V0-classification of the HDPE-composites was reached when Exolit AP 422 and RP were used together (20% APP and 10% red phosphorous).

The vertical UL-94 test checks only whether a material can be ignited with a small flame and thus investigates the starting phase of a fire. Using the cone calorimeter, more advanced fire stages can be simulated because the sample is exposed to a radiant heat flux which mimics a larger fire [31]. Results obtained with the cone calorimeter by Schubert et al. [32] showed that the combination of APP and expandable graphite increased the amount of residue formed compared to when either of the two FR was used separately. In addition, the total heat emitted could be reduced. For PP as matrix in WPC, best overall results were observed for flame-retardant mixtures based on 15 wt% EG or EG/APP combinations at different ratios [10]. Yin et al. [10] also concluded that adding RP and aluminum diethylphosphinate (AlPi) to EG exploits the combination of the strong flame-retardant action of EG in the condensed phase with flame inhibition.

In the present project, even though it could be shown that 20–30% of APP were sufficient to achieve V0-classification for a 0.8 mm sheet based on HDPE and wheat straw, this did not implicate that C or even B classification (EN 13501-1) for the co-extruded profiles could be reached. Hence, it was decided to use a larger amount of APP (40%) in the extrusion trials to ensure that a high level of fire-retardancy could be obtained.

### 2.2. Comparison of Hollow-Core and Solid Profiles with Fire-Retardants in the Co-Ex-Layer (Series 1)

#### 2.2.1. Limiting Oxygen Index (LOI), UL-94 Tests Fire Behavior

**Figure 3:**
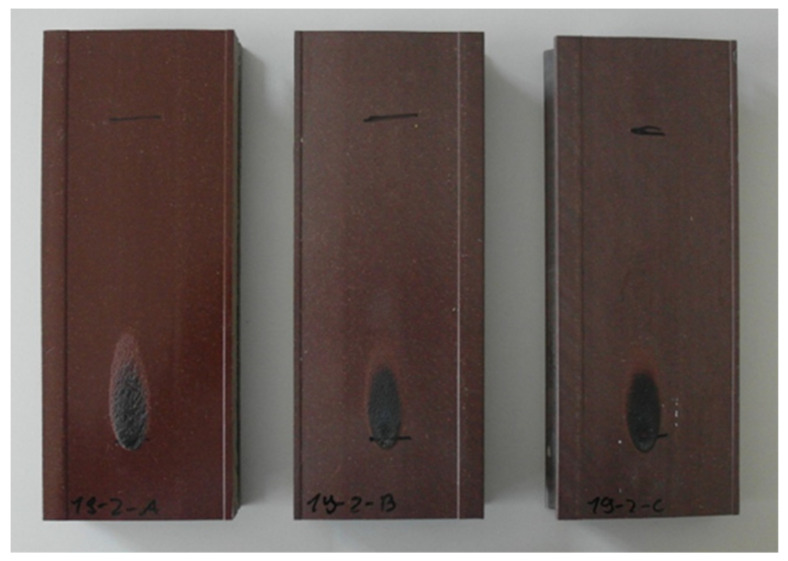
Hollow core profiles after the single flame source test (EN ISO 11925-2). From left to right: reference without FR, profiles with Budit 669, and profiles with FR CROS 490. Horizontal line on profiles indicates 150 mm-mark (vertical flame spread within 60 s after start of the flame treatment shall not be more than 150 mm).

**Figure 4:**
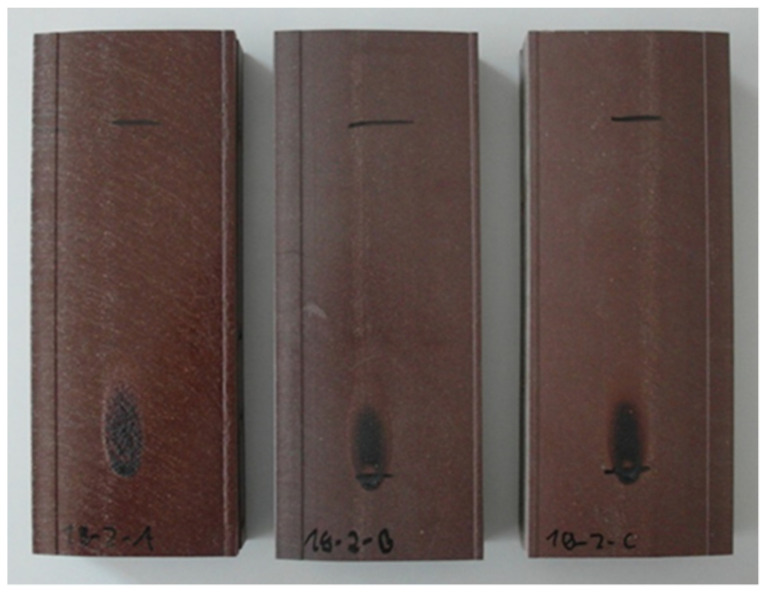
Solid profiles after the single flame source test (EN ISO 11925-2). From left to right: reference without FR, profiles with Budit 669, and profiles with FR CROS 490. Horizontal line on profiles indicates 150 mm-mark (vertical flame spread within 60 s after start of the flame treatment shall not be more than 150 mm).

A relatively high limiting oxygen index (LOI) with values of 32–34% was determined for the compounds used in the co-extruded layer, which indicates that the material is self-extinguishing (Table 2). In addition, small segments were cut from the profiles and tested in a modified, vertical UL-94 set-up. The results were V0 at 13 mm profile width with either of the two types of APP tested (Budit 669 and FR CROS 490). Next, the single-flame source test was performed with larger samples of 230 mm length as described in DIN EN ISO 11925-2. Sample width (100 mm) and thickness (25 mm) were different from the standard procedure since the profiles were used without modification. During each test, the flame was immediately self-extinguished, irrespective of if a fire-retardant was included or not (Figure 3 and Figure 4), which is similar to what has been found for composites with wood flour and recycled PP [33]. With a flame treatment of 30 s, vertical flame spread was well below 150 mm, which would classify the profiles in any of the European classes D, C, or B. However, to fully classify the profiles, a single-burning item (SBI) test would need to be performed.

**Figure 5:**
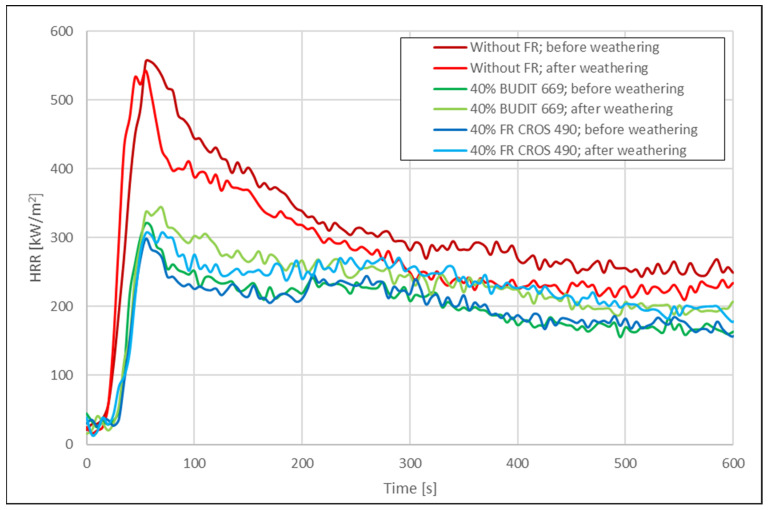
HRR of co-extruded hollow-core profiles before and after artificial weathering according to DIN EN ISO 4892-2 (cycle 2).

**Figure 6:**
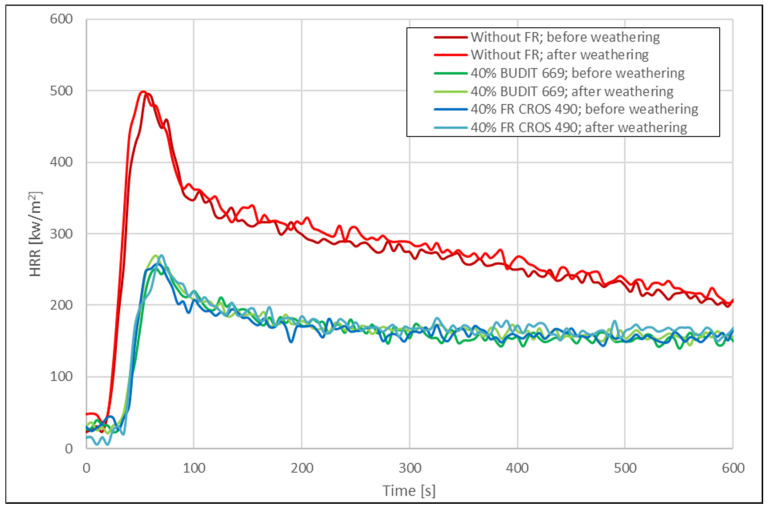
HRR of co-extruded solid profiles before and after artificial weathering according to DIN EN ISO 4892-2 (cycle 2).

Selected results from the cone calorimeter measurements (time to ignition, pHRR, THR_600s_, and mass loss) are shown in Table 3. Results for the HRR are shown in Figure 5 and Figure 6.

The values for the pHRR obtained in the present study are similar to results obtained by Schubert et al. [32] for extruded wood flour-based WPC with HDPE matrix and 15% APP (251 kW/m^2^); however, Schubert et al. used much thinner samples (pressed panels) of 6 mm thickness and a much lower amount of APP in their formulation, which makes it impossible to compare the results directly. It is well known that cone calorimeter results are highly dependent on the sample thickness [34]. In general, thicker samples display lower pHRR. Another difference between the work by Schubert et al. and this research is that Schubert used the FR for mass-protection of the composite and not only in the co-extruded layer.

In another recent publication, Yin et al. [10] subjected injection-moulded, FR-treated specimens to cone calorimeter measurements, in this case, based on PP as matrix and with 30% wood filler. In their investigations, the pHRR was relatively high when 20% APP were used as FR (326 kW/m^2^). However, when combinations such as 10% APP and 10% EG were used, much lower pHRR was achieved (189 kW/m^2^), which indicates that EG appears to be a suitable synergist for APP. The use of EG alone resulted in a pHRR of 152 kW/m^2^. One apparent disadvantage could be the relatively dark color of the EG.

Compared to solid wood, WPC generally shows higher pHRR. Wang et al. [35] presented data for the burning behavior of APP-treated plywood. Poplar veneers were immersed in aqueous solutions of APP with different concentrations. The pHRR of a control plywood (9 mm thickness) was 165 kW/m^2^ at an external heat flux of 35 kW/m^2^. With 20% of APP solution, pHRR was reduced to 83 kW/m^2^, hence, about half the value of untreated plywood. Even though the heat flux was lower compared to our study (50 kW/m^2^), it is obvious that straw-HDPE composites are more difficult to fire-protect than traditional wood composites. It also has to be considered that the tested plywood samples were far thinner than the profiles tested in this project (25 mm).

In most publications with cone calorimetry data for WPC, the curves for the HRR show a characteristic double peak that has not been observed in our experiments, possibly due to the thermal instability of the formulations with wheat straw. Usually, the first peak occurs shortly after ignition, with the height of the peak indicating a measure of the flame spread. The sharp increase of the first peak can be attributed to combustion of volatiles released from the materials surface. If fire-retardants are used, the pHRR is decreased. A second peak is often formed in lignocellulosic substrates as a result of char-surface cracking and combustion. The THR during the cone calorimeter run represents the integral of the HRR over time, i.e., the total heat output up to a specific point in time. The THR at the end of the test is the total heat emitted and therefore represents the fire load of the specimen in the fire test scenario [34].

**Figure 7:**
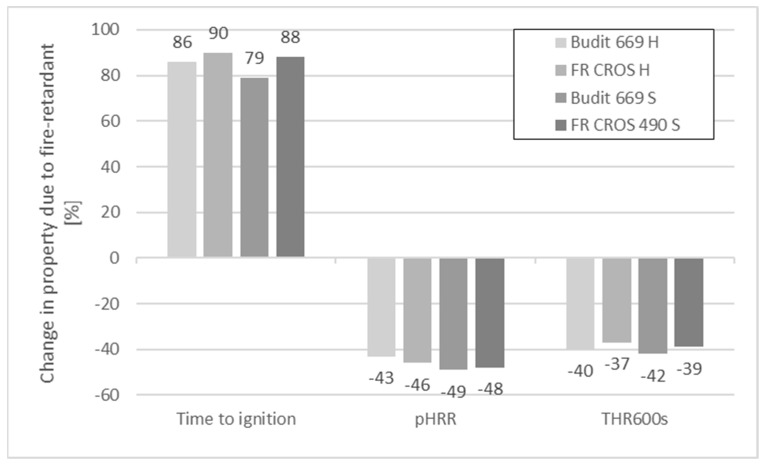
Change in time to ignition, peak heat release rate, and total heat release after 600s due to the fire-retardants in co-extruded profiles (before weathering). H: hollow-core profile; S: solid profile.

**Figure 8:**
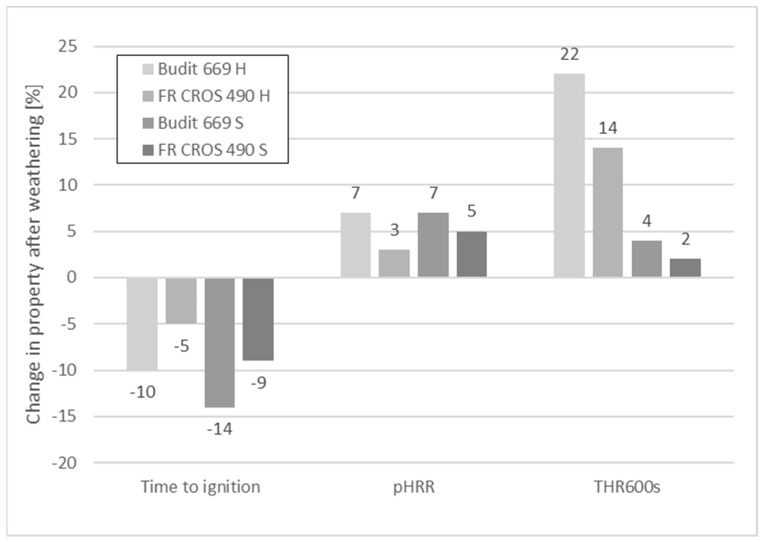
Change in time to ignition, peak heat release rate, and total heat release after 600 s in co-extruded profiles after artificial weathering for 28 days. H: hollow-core profile; S: solid profile.

The data for the samples before and after weathering were analyzed in Figure 7 and Figure 8. Before weathering, time to ignition (TTI) for the profiles without FR was 21 s for the hollow-chamber profiles and 24 s for the solid profiles. When FR were included in the formulations, TTI was extended by up to 90%, with FR CROS 490 showing slightly better performance than Budit 669. The pHRR was reduced by 43–49% due to the use of the FR, resulting in values of 298 kW/m^2^ for hollow-chamber profiles (with FR CROS 490) and 252 kW/m^2^ for full profiles (with Budit 669). However, it has to be noted that the reference without APP contained 40 wt.% more HDPE than the formulations including APP. This means that the potential fire load in the reference samples was higher. In the next series of formulations (series 2), a placeholder consisting of non-flammable calcium carbonate was included when APP was omitted to allow for better comparability.

Regarding THR after 600s, Figure 8 shows that this parameter was also strongly reduced due to the FR. The reduction was between 37% and 42%. As could be expected, mass losses were higher for the samples without FR and for the hollow-chamber profiles. The lowest mass losses were observed for the full profiles with FR (10–11% mass loss). It appears that the full profiles were more resistant to thermal degradation.

Mass losses were lower for the solid profiles, probably due to the higher mass per volume of material available compared to the hollow profiles. Without FR, mass losses were 36% for the hollow profiles and 15% for the solid profiles. When FR was included in the hollow profiles, mass losses were only 19% with Budit 669 and 25% with FR CROS 490. In case of the solid profiles, the corresponding values were 10% with Budit 669 and 11% with FR CROS 490. Overall, FR CROS 490 showed slightly better performance than Budit 669. Budit 669 only showed lower increases in THR_600s_, while TTI and pHRR were higher.

**Figure 9:**
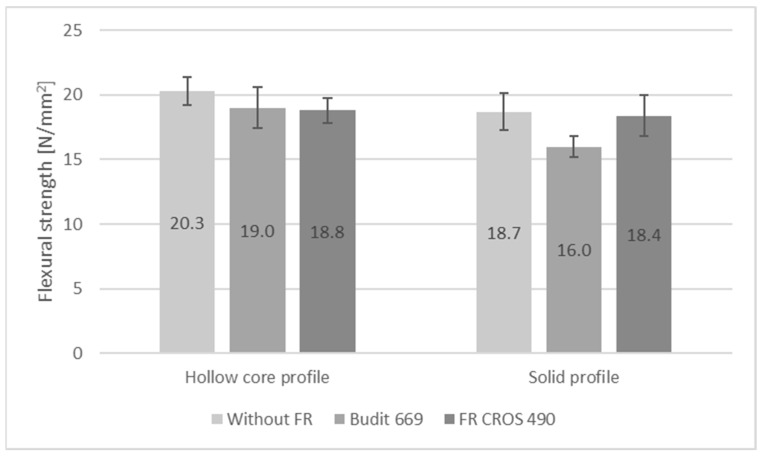
Flexural strength of co-extruded profiles with and without fire-retardants. No requirement provided in DIN EN 15534-5. Test performed according to EN 310 with support span of 500 mm and test specimen length of 600 mm.

To analyze the effects of 28 days artificial weathering, the result for each type of profile before weathering was regarded as reference (100%). For example, the hollow-core profiles with Budit 669 showed 10% shorter TTI and 7% higher pHRR after weathering (Figure 9). It can be seen that the differences between profiles with and without FR are more pronounced than the differences before and after weathering. This was especially the case for the full profiles. The hollow chamber profiles appeared more fragile due to their geometry, which may lead to lower stability when exposed to the flame. Overall, the largest differences before and after artificial weathering were observed for THR_600s_ of the hollow chamber profiles. The increase in THR_600s_ was 22% in the case of Budit 669 and 14% in the case of FR CROS 490. In case of the solid profiles, THR_600s_ after weathering was much lower than the hollow profiles (4% increase with Budit 669 and 2% with FR CROS 490). This can be explained by the differences in the profile geometries. In a hollow-shaped geometry, the amount of combustible material is lower and the fire load is reduced; however, fire propagation is increased [36]. Hence, after 600s, more heat will be released compared to solid profiles.

Many studies on the ageing of flame-retarded polymers have been performed; however, in most cases, fire tests were not carried out and wood or natural fibers were not included [37]. The effect of ageing on flame retardancy has been investigated only in few studies. Ageing encompasses many aspects such as thermal ageing, ageing in the presence of water and moisture, UV exposure, radiation, and recycling. Chen et al. [38] subjected PLA-ramie biocomposites to several days of weathering combining UV, heat, and water/moisture. They observed a strong decrease of LOI and UL-94 rating of the composites after weathering. It appeared that water and not only UV irradiation played a role in the deterioration of the fire performance. Migration of APP to the surface is assigned to its hydrolysis because APP is initially water-insoluble and becomes soluble when its molecular weight decreases. Migration may also lead to a heterogeneous distribution of APP into the material. In addition, the coverage of fibers by the FR is likely reduced after weathering which may promote a candlewick effect. In general, the synergistic effect of UV radiation and water seems to be well established. Braun et al. [39] investigated the weathering resistance of halogen-free flame retardance in thermoplastics, i.e., polycarbonate blends, polyamide, and PP. For the protection of PP, an intumescent formulation based on APP was used. It was shown that in this case, fire-retardancy is dominated by a surface mechanism and depends on the duration of weathering. A worsening in the formation of the intumescent network was observed; however, the material was still very effectively flame-retarded compared to unprotected PP. In general, it was demonstrated that the influence of weathering on the fire behavior was small compared to the differences between fire-retarded and non-fire-retarded materials. This conclusion applies to the results obtained in the present study for composites with HDPE and wheat straw also.

Almeras et al. [40] investigated the effects of artificial weathering and recycling on intumescent PP-based blends. It was shown that APP is degraded into ortho-, pyrophosphate, and short-chain polyphosphates after artificial weathering. These modifications resulted in loss of the ammonium contents and decrease in fire performance. In addition, the effects of recycling were examined and simulated by a multi-extrusion process. Changes induced by recycling were compared to the effects of ageing.

The charring agent tris(2-hydroxyethyl) isocyanurate (THEIC) in combination with APP was used as intumescent flame retardant for PP by Chen et al. [41]. They performed a water resistance test and soaked their composites in water at 70 °C for 36 h. This treatment revealed poor flame retardant durability under the parameters of their study and was explained with the high water solubility of THEIC.

#### 2.2.2. Evaluation of Durability Based on ETAG 028 (2012)

To evaluate the results of the artificial weathering tests in the present study, ETAG 028 (2012) was used. This ETA Guideline covers paints, coatings, varnishes, and surface impregnations intended to improve the reaction-to-fire performance characteristics of a surface of a construction product. Although co-extruded products are not specifically mentioned, ETAG 028 was applied since a co-extruded layer is similar to a thick coating. Encapsulation coating systems are covered by ETAG 028, i.e., coating systems that when applied completely encase a surface to a thickness of at least 1 mm.

The following end use categories are defined in ETAG 028 (2012) in relation to environmental conditions:

- Type X—Fire retardant products intended for all conditions (internal, semi-exposed, and exposed)
- Type Y—Fire retardant products intended for internal and semi-exposed conditions. Semi-exposed includes temperatures below zero, but no exposure to rain and limited exposure to UV (but UV is not assessed)
- Type Z1—Fire retardant products intended for internal conditions (excluding temperatures below zero) with high humidity
- Type Z2—Fire retardant products intended for internal conditions (excluding temperatures below zero) with high humidity classes other than Z1

For the co-extruded façade profiles in the present project, type X was applied.

According to ETAG 028, durability of fire performance is assessed by subjecting test specimens to fire tests in accordance with ISO 5660-1:2002, then further specimens are subjected to ageing procedures and fire tested again using the same method (cone calorimeter). After undergoing the ageing tests, the averaged fire performance of the test specimens shall fulfil the requirements provided in Table 4. The specific ageing (weathering) regime to be applied is described in Annex B of ETAG 028. For type X products, the test samples shall be subjected to a 24 h exposure cycle consisting of 4 h wetting, 4 h drying, 4 h wetting, 4 h drying, and 8 h rest. This cycle shall be repeated for a total of 1000 h (6 weeks). The weathering regime used in the present project deviated from this protocol, as outlined in the materials and methods section. An important aspect is that all specimens shall be conditioned to constant mass before testing in the cone calorimeter.

The performance requirements for durability of fire-retardant wood products listed in ETAG 028 are also listed in DIN EN 16755. If requirements are met, products can be marked with DRF (durability of reaction-to-fire performance) class symbols. In the case of façade claddings, used in exterior conditions, the symbol DRF class EXT would be applicable.

The individual values for RHR_30save_ were calculated for the weathered, co-extruded profiles (Table 5). RHR_30save_ was calculated as the sum of the HRR peak value and three values before and after the peak (seven values in total) divided by seven (DIN EN 16755). Hence, RHR_30save_ is always lower than the pHRR. The values for the RHR_30save_ were then compared with the requirements given in Table 4. It can be seen that for each of the four profiles tested, the values for RHR_30save_ were higher than 220 kW/m^2^, which means that neither classification C nor B according to EN 13501-1 was reached. At best, class D was reached. The best result in terms of the RHR_30save_ (242 kW/m^2^) was obtained for the full profiles with FR CROS 490, and this value was almost at the threshold to reach C classification. Considering that ∆THR_600s_ was very low (only +2%), it can be concluded that for this type of profile, class C could potentially be reached following some optimization. With the exception of the hollow chamber profile including Budit 669, all profiles reached the requirement for ∆THR_600s_, i.e., the increase was less than 20% compared to the value for THR_600s_ before weathering.

Overall, the results shown in Table 5 were better for the full profiles than for the hollow-chamber profiles. This agrees with results reported by Seefeldt and Braun [36], who investigated the effects of profile geometry on the burning behavior of wood-plastic composites in depth using the cone calorimeter. According to their results, the cavities in hollow-chamber profiles cause thermal feedback that increases the HRR at the beginning of burning, compared to solid profiles. They concluded that fire propagation or fire spread for a hollow WPC profile is higher than for a solid profile.

#### 2.2.3. Color Changes after Artificial and Natural Weathering

Color changes of co-extruded profiles after artificial weathering in a xenon device and after natural weathering are shown in Table 6. In general, the geometry of the underlying profile (hollow chamber versus full profile) should not affect the weathering characteristics of the co-extruded cap layer. However, since there can be slight changes in processing parameters and distribution of additives, it was decided to subject both profile geometries to weathering and evaluate the results for the two types separately. As can be seen in Table 6, color changes were similar for both profile geometries. For example, the formulation without FR displayed a ∆E* of 3.22 after 300 h and 6.01 after 28 days for the hollow-chamber profile. The respective values for the full profile were 4.03 and 5.02. As could be expected, profiles without FR showed the lowest color change. When FR were included, color change increased to a maximum of 11.01 (hollow chamber profiles with Budit 669) and 13.47 (full profiles with Budit 669) after 28 days of weathering. The intumescent FR may have influenced the light stabilized compound. Most flame retardants influence the photooxidative stability of the polymer substrate directly through acceleration of the degradation process or indirectly with the antioxidants and light stabilizers [42].

Overall, in terms of color change, performance of the profiles with FR CROS 490 was slightly better compared to Budit 669, which may be due to the difference in the coating and composition of the two FR. At present, no requirement is provided in DIN EN 15534-5 regarding color change after artificial or natural weathering. Color changes shall only be recorded. However, a ∆E* of 10 or higher may be regarded as critical from an end user’s perspective since at this level the color of the profile will have changed noticeably. In DIN EN 15534-5, the duration for artificial (xenon) weathering of composites using EN ISO 4892-2 is set as 300 h, which is appropriate for formulation development but not to determine long-term color changes. For example, in the case of the full profiles with Budit 669, the ∆E* was 6.80 after 300 h but increased to 13.47 after 28 days. Without inclusion of a FR, ∆E* increased from 3.22 to 6.01 (hollow-chamber profiles).

In addition to artificial weathering, the profiles were also subjected to natural weathering. With the exception of one type of profile (Bu S), there was good agreement between the color change of profiles that had been exposed to xenon weathering for 28 days and to natural weathering for one year. In addition, Table 6 shows that there was only little further increase in color change between 6 and 12 months of natural weathering. Most of the color change occurred up to a weathering period of 6 months. Overall, the inclusion of the FR worsened the outdoor durability, and the observed color changes are too high. Usually, a ∆E* value of 1 or 2 is considered excellent. If the ∆E* value is smaller than 3, this indicates a color difference that is not detectable by the human eye [43]. The color stability of various fire-retarded WPC with and without light stabilizers was first investigated by Garcia et al. [17]. In this case, accelerated weathering was done in a QUV chamber for 300 h and 600 h. When APP without stabilizer was used, a high ∆E* value of 32 was obtained. Best durability results were obtained for stabilized WPC with aluminum hydroxide (∆E* value of 14.5 after 600 h).

Turku and Kärki [44] investigated the influence of aluminum trihydroxide (ATH), zinc borate, melamine, graphite, and titanium dioxide on the durability of PP-based, co-extruded WPC. After accelerated xenon weathering for 1000 h, it was found that the FR did not influence the photo-oxidation mechanism of the composites. The color change of the FR-treated samples was lower than of the non-FR-treated composites, which is in contrast to the results of the present study. However, in the work by Turku and Kärki, APP was not included.

#### 2.2.4. Mechanical Performance, Water Uptake and Swelling, and Thermal Conductivity

In addition to the reaction-to-fire performance, flexural strength properties, swelling, water absorption, and thermal conductivity of the profiles were determined since these properties are also relevant during service life of the profiles. Abu Bakar et al. [45] reported losses in flexural and tensile strengths of WPC based on PP and wood flour with fire-retardants. Stark et al. [46] report 20% reduced flexural strength of WPC with 60% wood filler and 10% of APP; in addition, flexural modulus of elasticity (MOE) was reduced by 24% due to APP. However, other FR such as zinc borate were found to increase strength and MOE.

**Figure 10:**
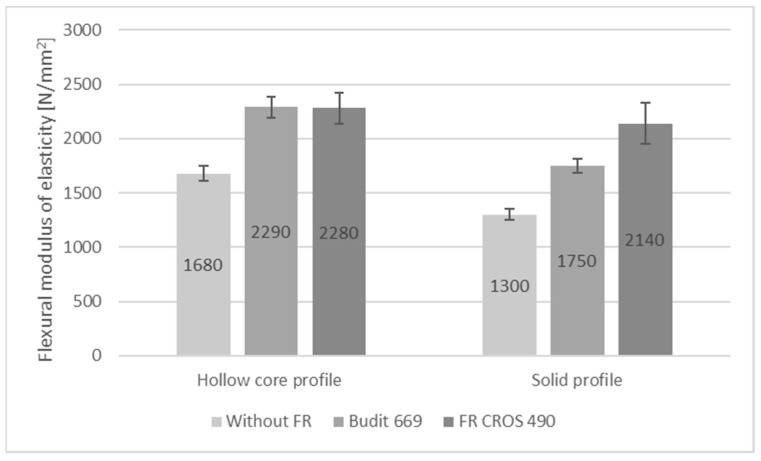
Flexural modulus of co-extruded profiles with and without fire-retardants. No requirement provided in DIN EN 15534-5. Test performed according to EN 310 with support span of 500 mm and test specimen length of 600 mm.

**Figure 11:**
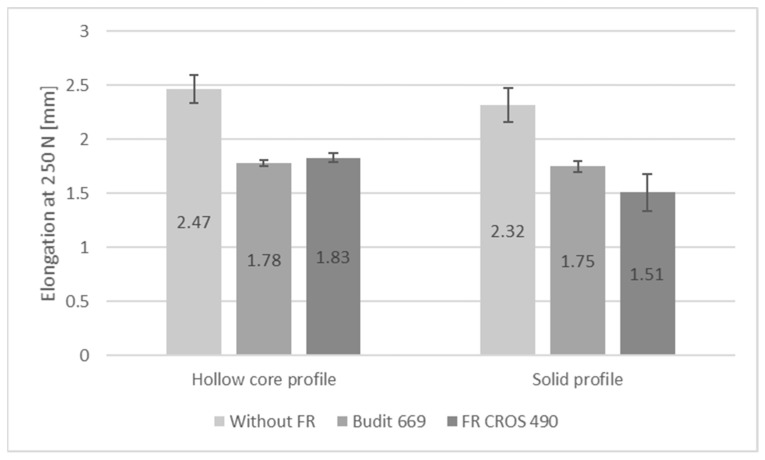
Elongation at 250 N of co-extruded profiles with and without fire-retardants. Requirement (DIN EN 15534-5): ≤5 mm. Test performed according to EN 310 with support span of 500 mm and test specimen length of 600 mm.

Results for flexural strength, modulus of elasticity, and elongation at 250 N are shown in Figure 9, Figure 10 and Figure 11. It can be seen that in the case of the hollow core profiles, flexural strength was only minimally reduced when FR were included. However, it has to be considered that for the reference formulation, the amount of HDPE was higher when the FR was omitted, while the amount of wheat straw was kept constant.

In case of the solid profiles, flexural strength was reduced from 18.7 N/mm^2^ for the reference profiles without FR to 16 N/mm^2^ for the profiles with Budit 669. There was no reduction in flexural strength with the use of FR CROS 490. Since the FR acts as an additional filler, flexural modulus of elasticity was significantly increased with Budit 699 and FR CROS 490. For the hollow core profiles, the increase in MOE was practically identical for both FR (from 1680 N/mm^2^ to 2290 or 2280 N/mm^2^), whereas for the solid profiles, the use of FR CROS 490 lead to a higher MOE compared to Budit 669, which could be due to the FR composition or differences in distribution of the FR in the polymer matrix. In terms of flexural strength properties, a requirement according to DIN EN 15534-5 exists only for elongation at 250 N (<5.0 mm for the arithmetic mean value), which was fulfilled by all profiles tested (Figure 11). As expected, profiles with FR and therefore higher filler content showed lower elongation (approximately 25–30% lower).

**Figure 12:**
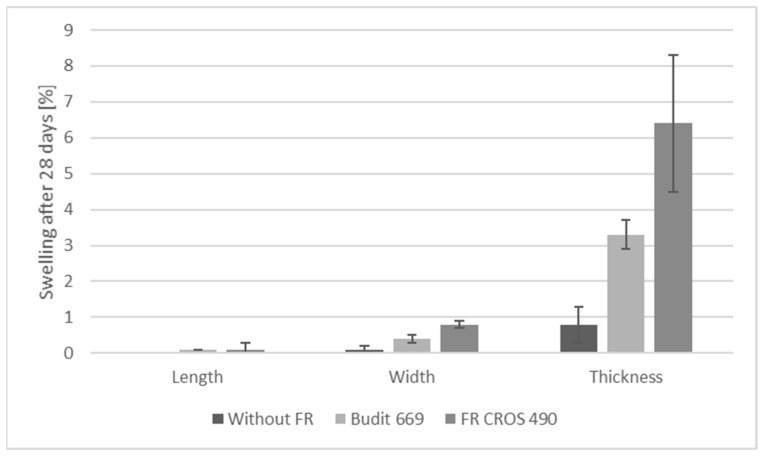
Swelling of co-extruded hollow core profiles after 28 days storage in cold water. Requirements according to Table 7 were fulfilled.

**Figure 13:**
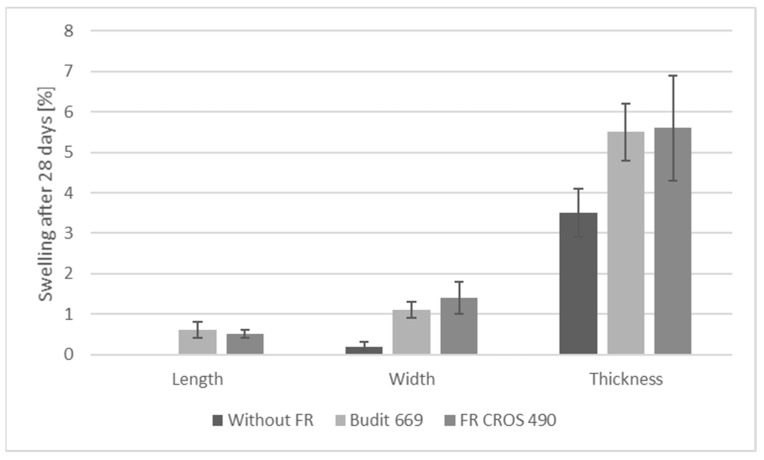
Swelling of co-extruded solid profiles after 28 days storage in cold water. Requirements according to Table 7 were fulfilled.

**Figure 14:**
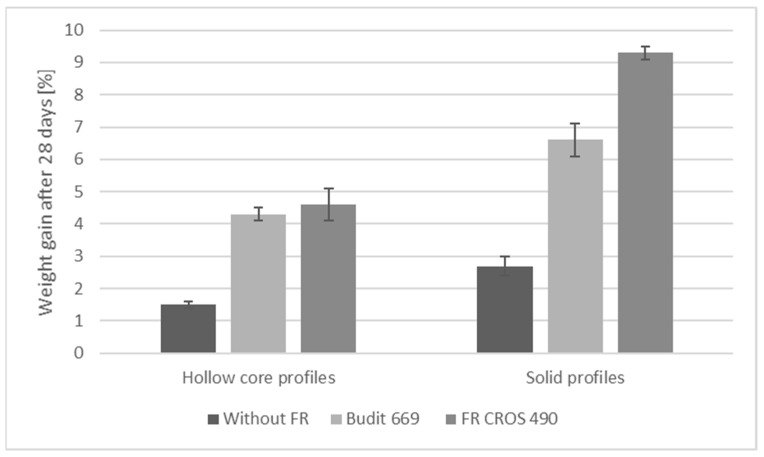
Weight gain of co-extruded profiles after 28 days storage in cold water. Requirements according to Table 7 were fulfilled, with the exception of the average for the solid profiles with FR CROS 490.

The requirements for swelling and water absorption are summarized in Table 7, and results are shown in Figure 12, Figure 13 and Figure 14. All criteria were met, except for the weight gain of solid profiles with FR CROS 490. Values for thickness swelling were highest, followed by the values for width and length swelling. Overall, swelling values for solid profiles were higher than for hollow chamber profiles, which is due to the higher weight of the solid profiles (approx. 1300 g/m) compared to the hollow chamber profiles (approx. 700 g/m). Weight gain was higher for the solid profiles, and the use of the FR lead to increased weight gain, which is probably due to the hygroscopicity of the FR. The highest weight gain (approx. 9% for solid profiles) was observed when FR CROS 490 was included. The weight gain with Budit 669 reached a maximum of 7%, which is acceptable. The differences in weight gain of the profiles by the two FR could be due to the differences in coating of the APP. The type of FR, including their coatings, will likely also affect the efficiency of the coupling agent (MAPE). Hence, water uptake and swelling were increased because bonding between the coupling agent, polymer matrix, and wheat straw may have been reduced.

Thermal conductivity λ of the profiles with FR CROS 490 was 0.13 W/(m∙K) for the co-extruded hollow-chamber profile and 0.21 W/(m∙K) for the co-extruded solid profile, with corresponding values for thermal transmittance (U-values) of 5.4 and 7.8 W/(m^2^∙K), respectively (Table 8). The higher thermal conductivity for solid profiles is due to the higher density of the material. The λ values are close to the values for solid spruce wood with 0.10–0.11 W/(m∙K) in grain direction and 0.22 W/(m∙K) parallel to the grain [47]. For comparison, thermal conductivity of high-pressure laminate (for example, Trespa Meteon: 0.3 W/(m∙K); [48]) or unfilled HDPE (0.38–0.51 W/(m∙K); [49]) is much higher. Based on thermal conductivity values, wheat straw-based, fire-retarded profiles are suitable for application as façade materials.

#### 2.2.5. Thermogravimetric Analysis (TGA)

**Figure 15:**
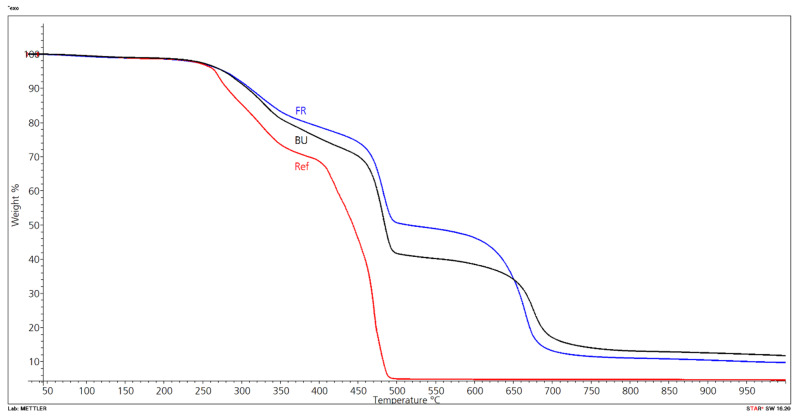
TGA of compounds with Budit 669 (BU), FR CROS 490 (FR), and without fire-retardant (Ref); composition of compounds shown in Materials and Methods.

The results for the three compounds with Budit 669, FR CROS 490, or without FR are shown in Figure 15. The formulation without FR shows two degradation steps, while thermal degradation of the compounds with FR occurs in three steps. The compound without FR starts to degrade earlier and shows higher mass loss. Degradation begins at about 200 °C, and maximum degradation temperatures in the first step are reached at 270 °C for the reference compound and at about 320–328 °C for the compounds with FR. According to the technical data sheet of Budit 669, this coated intumescent combination based on APP starts to decompose at temperatures of more than 275 °C. The decomposition temperature of Budit 669 is higher than of FR CROS 490, a long chain APP phase II that starts to decompose at a temperature of more than 250 °C. Above 300 °C, it decomposes to polyphosphoric acid and forms a gas layer that reduces the amount of oxygen available to the substrate. Subsequently, dehydration of polyols and carbohydrates occurs and a heat-protective barrier layer is produced by foaming (intumescence). It can be seen in Figure 15 that residual mass of the compound with FR CROS 490 is initially slightly higher because this FR is beginning to decompose and react earlier whereas Budit 669 starts to react slightly later. Hence, the compound with Budit 669 loses more weight in this initial phase.

Maximum degradation temperature of the second step is reached at about 480 °C for the two compounds with FR (Table 8). In this step, the highest mass losses occur (29–32%). Onset of the third degradation step is beyond 500 °C. Up to a temperature of approximately 650 °C, the mass loss of the compound with FR CROS 490 is lower than Budit 669. From 650 °C onwards, the compound with FR CROS 490 shows less thermal stability and degrades more, resulting in lower final residue (9.7% vs. 12.9%). The individual degradation steps with their respective onset and final degradation temperatures are shown in Table 9. The degradation steps can be assigned to the degradation of hemicellulose, cellulose, and lignin of the wheat straw and of the HDPE. The first degradation step corresponds to the wheat straw and the second one mainly to the degradation of the HDPE [5]. The main components of wheat straw are cellulose, hemicelluloses, and lignin. The thermal degradation temperatures of hemicelluloses, cellulose, and lignin are between 150 °C and 350 °C, 275 °C and 350 °C, and 250 °C and 500 °C, respectively [50]. Polyethylene degrades mostly in the temperature range between 489 °C (maximum weight loss temperature) and 520 °C [5]; hence, there is some overlap in the thermal degradation curves of wheat straw and HDPE. Thermal stability of HDPE- and PP-wheat straw bio-composites was analyzed in detail by Zabihzadeh [50]. In summary, in the present study, the use of FR CROS 490 resulted in higher thermal stability of the compound in the temperature range between 300 °C and 650 °C. Mass losses in steps 1 and 2 were lower for compounds with FR CROS 490 compared to Budit 669, whereas final residues were slightly higher for compounds with Budit 669.

It was found during profile extrusion of wheat-straw based formulations that the material burnt easily in the extruder and the processing window was very narrow compared to extrusion of wood particle-HDPE-based formulations. These differences are most likely due to the chemical composition and morphology of the lignocellulosic materials. Wood contains two to three times more lignin than wheat straw (Table 10). Lignin is seen to be the thermally most stable component of wood even though chemical changes occur already at temperatures below 200 °C [51]. The higher the lignin content, the higher the char yield and the better the flame retardancy performance [52]. Lignin requires more energy to pyrolyze and produce flammable fuel [53]. The resulting char layer has an insulating effect on the underlying material, preventing continuous burning. In the case of natural fibers, results from Dorez et al. [54] indicate that natural fibers with low lignin content such as flax and hemp display better fire behavior than fibers with high lignin content. It was shown by the authors that the presence of a low content of lignin and high content of cellulose affects the degradation pathway of cellulose. This leads to charring and incomplete combustion of these fibers and limits their contribution to the heat evolved during burning.

**Figure 16:**
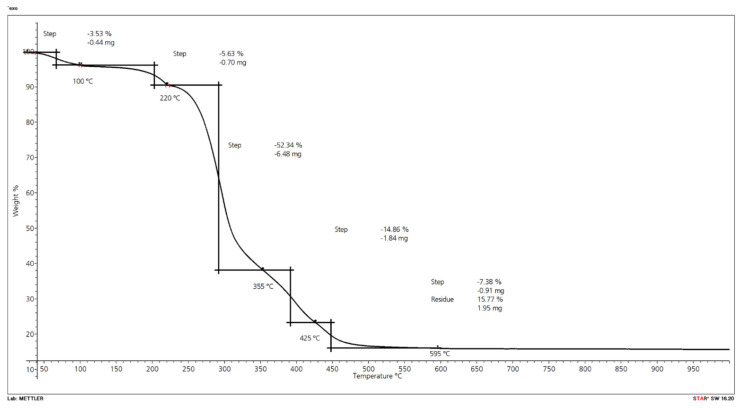
Thermal degradation steps for wheat straw particles.

**Figure 17:**
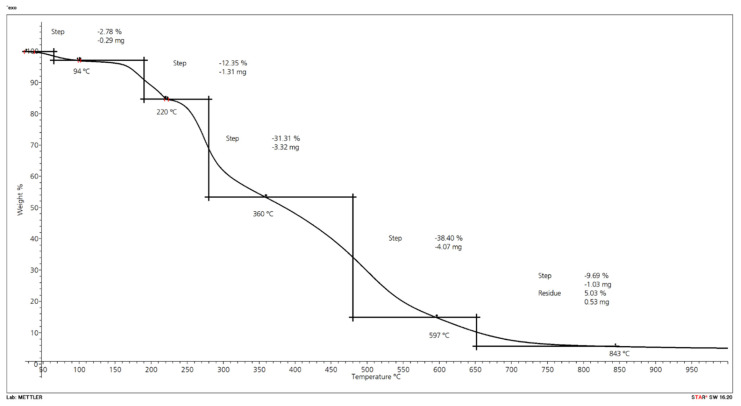
Thermal degradation steps for wood flour (Arbocel C320).

In the present study, the comparison of wood and wheat straw of a comparable particle size showed that wood flour is thermally more stable, with weight losses of 32% during the first degradation step compared to 53% for the wheat straw (Figure 16 and Figure 17; Table 11). This first step represents the temperature zone, which is relevant for the processing of straw in extrusion. During the second step, beyond approximately 360 °C, mass loss of the wood particles was higher (39% compared to 15% for wheat straw). Zhang et al. [5] determined the thermal stability of composites based on recycled PE and wheat straw of different fiber sizes (long, medium, and short). They found that the particle size of the wheat straw had no significant influence on the thermal stability of the composites. In their study, they did not compare wheat straw and wood fibers in terms of thermal stability.

In the present project, the final residue of the wheat straw (15.8 wt.%) was three times higher than wood particles (5 wt.%), which can be attributed to the high amount of silica in wheat straw. While wheat straw contains 6% of silica, wood contains only up to 0.001% of silica (Table 12). Despite the decreased thermal stability of wheat straw compared to wood over a wide temperature range, the final residue is much higher. The silica is present in a thin layer at the outer surface of the epidermis, which may inhibit bonding with certain matrix polymers [6]. Chemical treatments to improve surface adhesion can be done or, alternatively, mechanical crushing of the straw has been performed.

Pasangulapati et al. [55] investigated the effects of cellulose, hemicellulose, and lignin on the thermochemical conversion characteristics of wheat straw, eastern redcedar, and other biomass materials. In their work, no significant difference was found in the weight loss profiles of wheat straw and eastern redcedar, although their cellulose, hemicellulose, and lignin contents were considerably different. In contrast to the present study, Pasangulapati et al. [55] used argon, an inert gas, during the TGA measurements, which may partially explain the observed differences. In the present investigation, measurements were done under air flow to simulate the conditions in practice during extrusion.

### 2.3. Comparison of Profiles with Fire-Retardants in the Co-Ex-Layer or in the Bulk of the Profiles (Series 2)

#### 2.3.1. Cone Calorimeter Results and Evaluation Based on ETAG 028 (2012)

In series 2, profiles with the FR in the co-extruded layer only or in the bulk of the profile were compared. In this series, only solid profiles were extruded. The viscosity of the HDPE type, which was used for bulk profile extrusion, was too high for processing of the co-extruded layer; hence, a different HDPE with low viscosity was chosen. Based on the results from series 1, FR CROS 490 was chosen as FR because it had performed slightly better in terms of the cone calorimeter data and regarding color stability after weathering. Calcium carbonate as non-flammable filler [56] was used to determine if improvement of the fire performance was due to the use of the FR or due to the reduction in HDPE content. Calcium carbonate decomposes at 825 °C to generate solid calcium oxide and gaseous carbon dioxide [31]. Both products do not support combustion.

**Figure 18:**
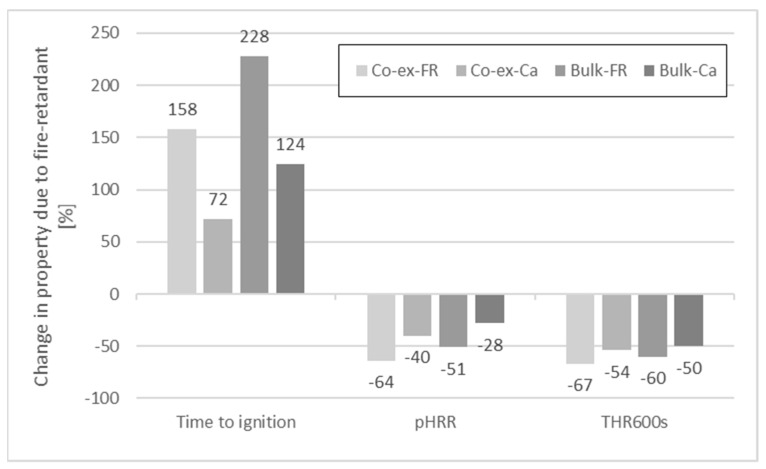
Change in time to ignition, peak heat release rate, and total heat release after 600s due to the fire-retardant (FR CROS 490) or due to calcium carbonate (Ca) in solid profiles (before weathering). FR CROS 490 or Ca were added either in the co-extruded layer or in the bulk of the profiles.

The results for the cone calorimeter measurements are shown in Table 12. Before artificial weathering, pHRR, THR_600s_, and time to ignition could be significantly reduced due to the incorporation of the FR (Figure 18). Without FR, time to ignition was 18 s for the co-extruded profile and 23 s for the uncapped profile. When the FR was included, time to ignition was increased by 158% (co-extrusion) and 228% (uncapped). Calcium carbonate also increased TTI but not to the same extent as the FR. The pHRR was reduced by 64% and 51% for co-extruded and uncapped profiles due to the FR. Use of the calcium carbonate also reduced the pHRR, but again, not to the same extent as the FR. THR_600s_ was reduced by 60–67% when FR CROS 490 was included and by 50–54% when calcium carbonate was used.

**Figure 19:**
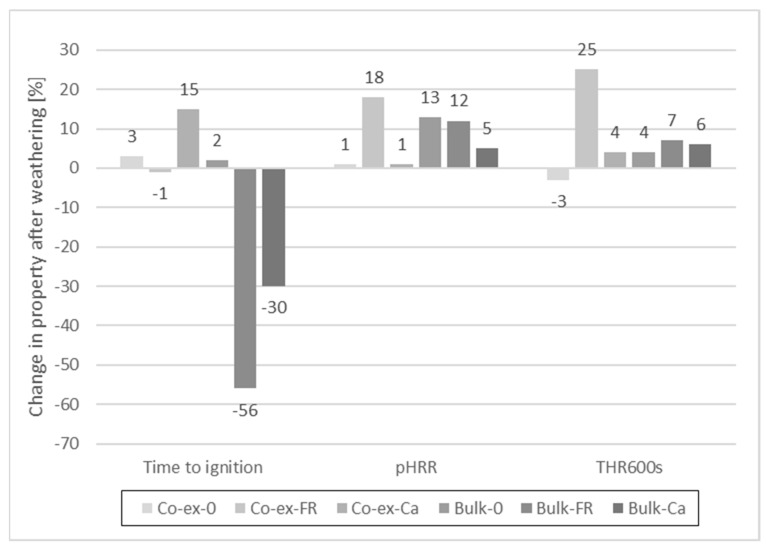
Change in time to ignition, peak heat release rate, and total heat release after 600s in extruded profiles with fire-retardant (FR CROS 490) or calcium carbonate (Ca) or without any filler (0), after artificial weathering for 28 days. FR CROS 490 or Ca were added either in the co-extruded layer or in the bulk of the profiles.

After articifical weathering for 28 days (Figure 19), time to ignition was hardly affected for the co-extruded profiles, irrespective if the FR was used or not. A slight increase in TTI (15%) was observed with CaCO_3_ as filler. However, in bulk-protected profiles, TTI was noticeably reduced after weathering when either the FR (−56%) or CaCO_3_ (−30%) were included. There was hardly any change in TTI after weathering when no filler was included at all.

**Figure 20:**
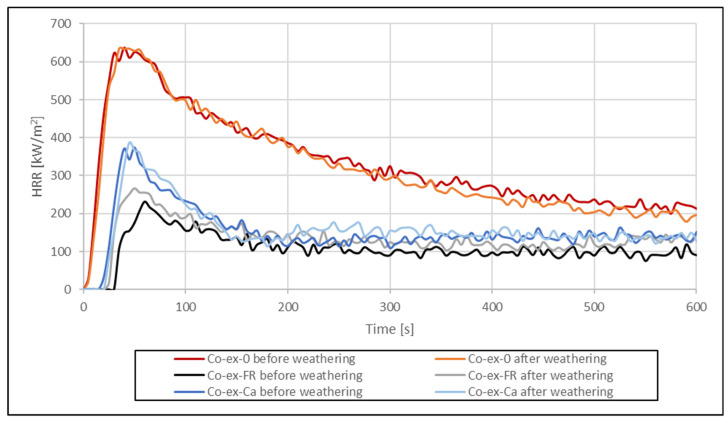
HRR of profiles with co-extruded layer before and after weathering.

**Figure 21:**
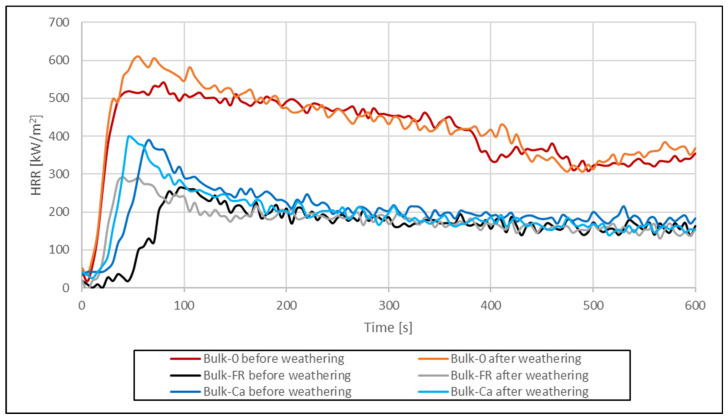
HRR of profiles with addition of FR CROS 490 or calcium carbonate in the bulk, before and after weathering.

As could be expected, artificial weathering increased the pHRR (Figure 20 and Figure 21) to some extent but the effect was less compared to the results with and without FR. The curves for the HRR of co-extruded profiles show little variation before and after weathering. The curves for the bulk-protected profiles show more pronounced differences. Here, pHRR for the weathered profiles was either higher compared to non-weathered profiles, or time to ignition was shorter. An explanation for the observed differences between co-extruded and bulk-protected profiles could be the differences in viscosity of the HDPE used.

The comparison of the HRR shows that the curves for profiles with CaCO_3_ are between the curves with and without FR, which indicates that CaCO_3_ contributes to decrease the HRR, albeit to a much lower degree than the FR. CaCO_3_ is often used as low-cost filler in thermoplastics, and since it is non-flammable, it dilutes the total amount of fuel to be consumed [56]. In this regard, it is considered as a mineral filler flame retardant. However, its effectiveness in reducing the pHRR is lower than for other mineral flame retardants. Rothon and Hornsby [57] compared the results for aluminum hydroxide and calcium carbonate at equivalent weight loadings of 60% in an ethylene vinyl acetate copolymer. While pHRR of the formulation with aluminum hydroxide was 125 kW/m^2^, pHRR was 310 kW/m^2^ with calcium carbonate (at 35 kW/m^2^). The corresponding UL-94 classifications were V0 and unclassified, respectively.

For PP as matrix, it was shown that calcium carbonate nanoparticles in combination with 5 wt.% montmorillonite reduced the pHRR by 53% [58]. Calcium carbonate has also been investigated in combination with APP to protect PP [59]. It was demonstrated that the two additives react to form ß-calcium metaphosphate with the evolution of ammonia, water, and carbon dioxide. Hence, the degradation of PP was delayed due to the formation of the barrier from the metaphosphate. The presence of the non-combustible gasses diluted the flammable gasses and reduced burning of the PP.

Isitman et al. [60] investigated the influence of calcium carbonate on the fire retardancy of intumescent PP composites. When calcium carbonate was added into the intumescent formulation (either based on a mixture of APP and PER, or based on surface-modified APP), the flame-retardant properties deteriorated.

Nikolaeva and Kärki [9] investigated the effects of various FR on the reaction-to-fire properties of co-extruded WPC with polypropylene as matrix and generally found similar values for the HRR as in the present study. The pHRR of 231 kJ/m^2^ for unweathered, co-extruded profiles with APP in our work is similar to the pHRR of 247 kW/m^2^ reported by Nikolaeva and Kärki, which was the lowest pHRR of all FR systems tested by them. However, the amount of APP used by Nikolaeva and Kärki in their formulation for co-extrusion was only 10%. Combinations of APP and natural graphite or expandable graphite increased pHRR slightly. However, at the same time, the TTI for the composite with APP was the shortest among the FR tested by them.

**Figure 22:**
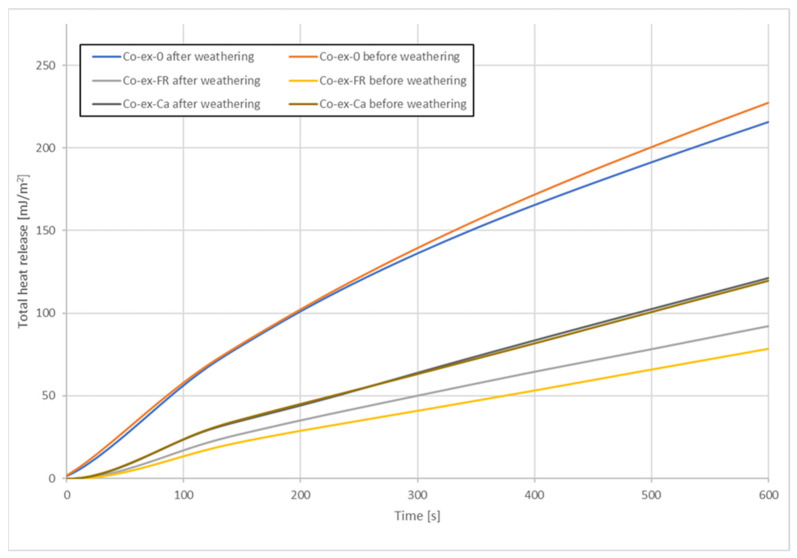
Total heat release for co-extruded formulations before and after weathering. 0: without any filler; FR: with FR CROS 490; and Ca: with calcium carbonate.

**Figure 23:**
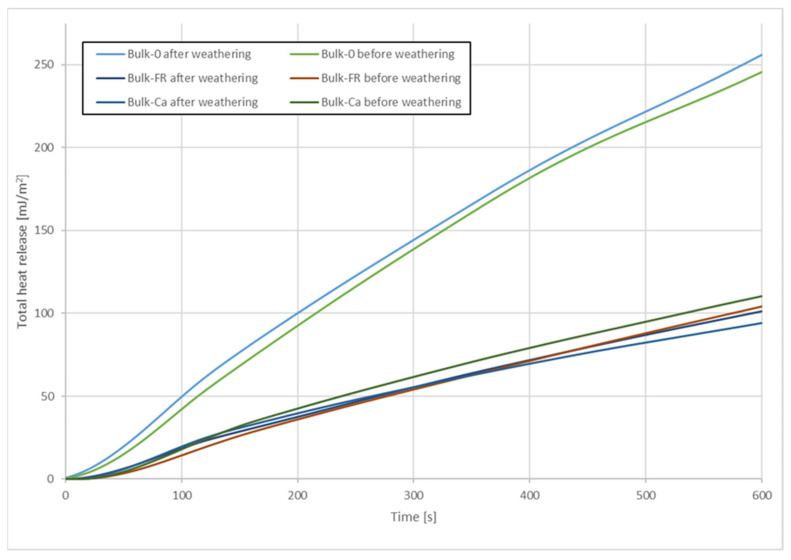
Total heat release for bulk-protected formulations before and after weathering. 0: without any filler; FR: with FR CROS 490; and Ca: with calcium carbonate.

The total heat released (THR) is shown in Figure 22 and Figure 23. THR increased linearly over time and reached much higher values for profiles without FR or with CaCO_3_. Lowest THR values were obtained with FR CROS 490 in co-extruded profiles. Artificial weathering increased THR for all profiles tested. Compared to the THR values shown by Nikolaeva and Kärki [9], the THR values in the present study are significantly higher, which could be due to the wheat straw filler and its lower thermal stability compared to the pulp cellulose filler used in their work.

As in series 1, the results obtained from the cone calorimeter measurements were evaluated based on requirements provided in ETAG 028 for RHR_30save_ and ∆THR_600s_. Table 13 shows that values for RHR_30save_ are always higher than the criteria to reach class C (220 kW/m^2^) or class B (150 kW/m^2^). The best value for RHR_30save_ was achieved with FR CROS 490 used in the co-extruded layer (241 kW/m^2^), which was relatively close to the threshold to reach class C. However, the value for ∆THR_600s_ (+25%) was too high (requirement: <20%). Values for ∆THR_600s_ for the other profiles in this series were all below 20% and would therefore meet the criterium for class B.

So far, classification of WPC with fire-retardants has been mostly done based on simulated FIGRA and SBI prediction. Generally, D classification has been reported [9,61]. We recently performed SBI tests with profiles based on either rice husks, wood flour, or wood flour pre-treated with FR. Profiles with rice husks, FR, and PP as matrix obtained D classification; with PVC as matrix for rice husks, C classification was obtained [62]. The combination of wood flour and HDPE resulted in C classification too. With a pre-treatment of the wood flour, B classification could be achieved.

#### 2.3.2. Color Changes after Artificial Weathering

Changes in color, lightness, and gloss after artificial weathering for 28 days are shown in Table 14. As in the first series, color changes were lowest when the FR was omitted. In case of the co-extruded profiles, ∆E* was 7.4, whereas in the case of the profiles with bulk protection, ∆E* was only 3.4. Hence, color change of co-extruded profiles without the FR was twice as high as that of the uncapped profile. The difference could be due to the type of HDPE used because different HDPE types were used in the co-extruded layer and bulk. In both types, antioxidants were used; however, the amounts and composition are not disclosed by the producer.

When APP was included, color change after weathering increased to 12 for the co-extruded profiles and to 30 for the bulk-protected profiles. Again, these differences can be due to the differences in the polymer or also due to variations in processing. The use of calcium carbonate as inert filler and placeholder for the FR also resulted in increased color change compared to when no APP was included (10.8 for co-extruded profiles and 13.3 for bulk-protected profiles). However, color change was lower than for fire-retarded profiles. In general, the values for color change of the profiles with either APP or calcium carbonate are very high. It was not the objective to optimize the formulations in terms of color stability; hence, UV stabilizers or pigments were not included in this series. It can be seen that the additives used in the first series (UV absorber, hindered amine stabilizer, and acid scavenger) are useful to maintain color stability of the fire-retarded profiles because in the first series lower values for ∆E* were obtained. On the other hand, it has to be considered that a different type of HDPE was used for the co-extruded layer in the second series.

#### 2.3.3. REM-EDX Analysis

Elemental analysis of the profile surfaces with FR before and after artificial weathering was determined by REM-EDX. The relative contents of carbon, nitrogen, phosphorus, silicium, iron, and oxygen were determined and the atomic percentages are shown in Table 15. Carbon is the predominant element with atomic percentages of 85–90%, followed by oxygen with 7–10% and further elements (P, N, Fe, and Si) in minor amounts. Since APP (FR CROS 490) has a P_2_O_5_ content of 68% and N content of 17%, both P and N contents are of primary interest. The profile co-ex-FR shows at.% for P of 0.8 (before weathering) and 1.6 (after weathering); hence, there appeared to be an increase in P content after weathering. This could be because different sets of samples before and after weathering were analyzed and due to local differences in the distribution of the FR on the profile surface. It can be assumed that leaching of P from the samples did not occur. The amount of N apparently also increased after weathering from 2.1 to 3.1 at.%. The decrease in carbon content from 89.7% to 85 at.% could be due to degradation of the substrate during artificial weathering, while oxidation of degradation products may have led to the increase in oxygen (6.6 at.% to 9.9 at.%). Due to the loss in carbon, there was an apparent increase in the relative amounts of P and N after weathering.

For the profile bulk-FR that contained FR CROS 490 in the bulk of the formulation, no difference in P content before and after weathering was determined. The amount of N was slightly reduced after weathering. In summary, neither for the co-extruded nor for the bulk-protected profiles was any significant leaching of the fire-retardants as measured in at.% for P and N observed, which is in agreement with the results from the cone calorimeter measurements before and after weathering.

## 3. Materials and Methods

### 3.1. Materials

The wheat straw in particle form (type: “Weizenstroh-Feinmehl”, delivered in bales) was obtained from company Cordes-Grasberg, Grasberg, Germany. It is primarily used for animal bedding and of high absorbing capacity. For comparison of thermal stability, wood particles (Arbocel C320, JRS, Rosenberg, Germany) were used.

A high-density polyethylene (HDPE) in powder form (56020 S, Total Petrochemicals, Brussels, Belgium) with an average particle size of 800 µm, density of 0.952 g/cm^3^, and a melt flow rate (190 °C, 21.6 kg) of 2.1 g/10 min was used as polymer matrix.

For the co-extruded layer in series 2, HDPE type 5502 (Total Petrochemicals) in pellet form was used. Density was 0.954 g/cm^3^, and melt flow rate (190 °C, 21.6 kg) was 22 g/10 min.

The coupling agent was a maleic-anhydride-grafted HDPE (MAPE; Scona TSPE 2102 GAHD, BYK-Chemie GmbH, Wesel, Germany) with a maleic anhydride content of 1.5% and melt volume-flow rate (MVR; 190 °C; 2.16 kg) of 1–4 cm^3^/10 min. Further additives were used as indicated in the formulations: a lubricant (blend of complex, modified fatty acid esters; TPW 113, Struktol, provided by Velux, Hamburg, Germany); a brown masterbatch based on inorganic pigments (74445F PE; Lifocolor, Lichtenfels, Germany); a synergistic processing and long-term thermal stabilizer with a high phenolic antioxidant content (Irganox B225, BASF Schweiz AG, Plastic Additives, Basel, Switzerland); a benzotriazole UV absorber (Tinuvin 326, BASF Schweiz AG, Plastic Additives, Basel, Switzerland); a mixture of oligomeric hindered amine stabilizers (Tinuvin 783, BASF Schweiz AG, Plastic Additives, Basel, Switzerland); and an acid scavenger based on magnesium/aluminium-hydrotalcite (Hycite 713, Clariant Produkte (Deutschland) GmbH, Moosburg, Germany). The following fire-retardants were used: a coated intumescent combination based on APP (Budit 669, Budenheim Ibérica, La Zaida (Zaragoza), Spain); APP phase II, coated with melamine, formaldehyde-free (FR CROS 490, Budenheim Ibérica, La Zaida (Zaragoza), Spain); uncoated APP (Exolit AP 422, Clariant Plastics and Coatings (Deutschland) GmbH, Frankfurt am Main, Germany); APP microencapsulated with melamine (Exolit AP 462, Clariant Plastics and Coatings (Deutschland) GmbH, Frankfurt am Main, Germany); a masterbatch with 60% red phosphorous in LDPE matrix (Masteret 10460 B2XF, Italmatch Chemicals S.p.A., distributed by WTH Walter Thieme Handel GmbH, Stade, Germany); expandable graphite (GHL PX 95 HT, Georg H. Luh GmbH, Walluf, Germany); and magnesium hydroxide (Magnifin H5-MV, Martinswerk GmbH, Bergheim, Germany). Where indicated, calcium carbonate (Omyacarb 40-VA, Omya GmbH, Cologne, Germany) was used as filler instead of FR.

### 3.2. Processing and Fractionation of Wheat Straw Particles

Particle size of the wheat straw as received was determined using a tumbling sieve with screen sizes of 3.15 to 5 mm; 1.25 to 3.15 mm; 0.6 to 1.25 mm; and <0.6 mm. Particles as received were used for the core of the profiles, whereas particles after sieving in a Condux mill with a 1 mm sieve insert were used for the co-extruded layers.

### 3.3. Compounding and Profile Extrusion

Fire-retarded materials for the co-extruded layer were developed by mixing the formulations in a kneading (batch) mixer with Banbury rotors (Thermo Fisher PolyLab with RheoMix 3000 QC, Karlsruhe, Germany). The resulting compound was milled into a fine powder and dried in an oven at 60 °C for 24 h. Compounds were pressed into thin sheets (0.8 mm thickness) at 185 °C using a hot press (Rucks Maschinenbau GmbH, Glauchau, Germany). Samples for UL-94 tests were cut from the sheets and conditioned at 20 °C and 65% relative humidity for one week prior to testing.

Samples for limited oxygen index (LOI) measurements were processed using a batch mixer, followed by mini-injection moulding (HAAKE Minijet II, Thermo Fisher Scientific, Karlsruhe, Germany) to prepare test specimens of 80 mm × 10 mm × 4 mm.

Compounds for profile extrusion were processed on a co-rotating twin-screw extruder (TSK 20, Theysohn Maschinenbau, Salzgitter, Germany). The four temperature zones of the compounder from feeder to the die exit were 200 °C, 185 °C, 110–130 °C, and 90–110 °C, depending on the formulation. The compound was processed into pellets in an underwater granulating system (EUP-ELG 50, ECON, Weisskirchen, Austria). Output was between 8 and 10 kg/h.

**Figure 24:**
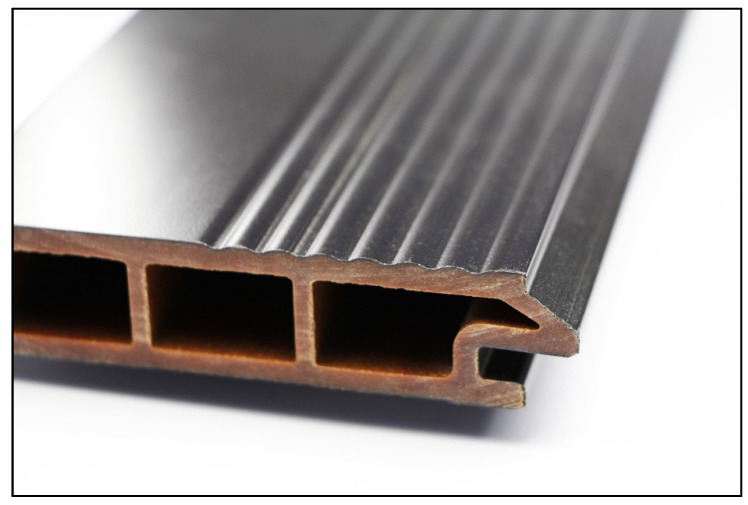
Profile geometry of the siding profile for extrusion.

Façade profiles (Figure 24) were extruded on a conical, counter-rotating twin-screw extruder (Battenfeld-Cincinnati MiniBex 54-C; 54 mm screw diameter). Temperature of the four zones from feeder to die were 150 °C, 155 °C, 160 °C, and 165 °C, with an output of 5–15 kg/h, depending on the type of profile (hollow-core vs. solid). A single-screw extruder (Göttfert X-Trude 600, Buchen, Germany) was used as co-extruder.

The first series of co-extruded profiles was processed as either hollow core (H) or solid (S) profile. In both types of profiles, the FR was present in the co-ex-layer only. Formulations are shown in Table 16. In this series, additives (Tinuvin 326, Tinuvin 783, and Hycite 713) were included to reduce the color change due to weathering and to determine the effects of artificial weathering on the fire-retarded, additivated profiles.

In the second series, co-extruded profiles and profiles without co-extruded layer were processed and compared. The FR was used either in the co-extruded layer or in the bulk of the profile (Table 17). In both cases, solid profiles were extruded. Different types of HDPE with either low or high MFI were used in the bulk of the profile or in the co-ex-layer to facilitate processing.

### 3.4. Limiting Oxygen Index (LOI) and UL-94 Tests

The LOI is the minimum concentration of oxygen, by volume percentage, in a mixture of oxygen and nitrogen at 23 ± 2 °C that will just support combustion of a material under specified test conditions (DIN EN ISO 4589-2). If a test specimen is hard to burn, more oxygen is required to cause flaming combustion and LOI is increased. The tests were done with a LOI analyzer (Dynisco, Franklin, MA, USA) using injection-molded specimens (80 mm × 10 mm × 4 mm).

UL-94 vertical (V) tests were performed based on IEC 60695-11-10 with 0.8 mm thick pressed sheets and with profile segments of 13 mm width (100 mm profile length).

### 3.5. Single-Flame Source Test

The single-flame source test was performed with a combustion chamber (BBB, G. Wazau Mess- und Prüfsysteme GmbH, Berlin, Germany) according to DIN EN ISO 11925-2 using a direct acting flame set at an angle of 45 °C to the vertical axis. Duration of the flame treatment was 30 s. According to DIN EN 13501-1, to reach classes D, C, or B, vertical flame spread within 60 s after start of the flame treatment shall not be more than 150 mm.

### 3.6. Cone Calorimetry

A cone calorimeter (Fire Testing Technology Limited, East Grinstead, UK) was used to evaluate the reaction-to-fire performance of the profiles (100 mm × 100 mm × 25 mm) according to ISO 5660-1-2015. External heat flux was 50 kW/m^2^. The samples were preconditioned at 20 °C and 65% relative humidity for at least one week before testing. Samples were tested in duplicate, unless measurements deviated by more than 10%. The key parameters determined were the heat release rate (HRR; kW/m^2^), peak heat release rate (pHRR, kW/m^2^), time to ignition (s), total heat release at 600 s (THR_600s_), mass loss (wt.%), and mass loss rate (g/s).

### 3.7. Thermogravimetric Analysis (TGA)

Thermal stability of the materials was analyzed using a thermal analyzer TGA/DSC 1 (Mettler-Toledo AG, Schwerzenbach, Switzerland). In case of the compounds, samples (approx. 10 mg) were heated from 25 °C to 1000 °C at a heating rate of 10 K·min^−1^ under a constant air flow of 50 mL·min^−1^. The same conditions were also used for analysis of the wheat straw and wood particles, except for the temperature range between 100 °C and 220 °C where a lower heating rate of 1 K·min^−1^ was chosen to allow for better differentiation between wheat straw and wood particles. This temperature range is particularly relevant for extrusion.

### 3.8. Thermal Conductivity

Thermal conductivity was determined according to DIN 12667 with a heat flow meter (HFM 300, Linseis GmbH, Selb, Germany). The test samples (profiles) were placed between a hot and a cold plate, and the heat flow between the plates was measured by two heat flux sensors. The heat transfer coefficient can be calculated from the measured heat flow through the sample divided by the cross-section area and the applied temperature difference. For a homogeneous material, the thermal conductivity λ is given by the quotient of heat transfer coefficient divided by the sample thickness. Because sample size for the test was 300 mm × 300 mm and only 100 mm profile segments were available, nine profile segments of 100 mm length in total were placed in the test equipment. Data for thermal conductivity λ were collected every 10 s, and 15 individual test runs were performed for each material.

### 3.9. Artificial and Natural Weathering

Artificial weathering was performed according to EN 15534-1 (2018) and EN ISO 4892-2 (2013), cycle 1, using a xenon device (Suntest XXL, Ametek, formerly Atlas, Linsengericht, Germany). According to cycle 1 in EN ISO 4892-2 (2013), the settings were 102 min of dry exposure followed by 18 min of water spray at an irradiance (300–400 nm) of 60 ± 2 W/m^2^. Conditions during the dry exposure were as follows: black-standard temperature: 65 ± 3 °C; chamber temperature: 38 ± 3 °C; and relative humidity: 50 ± 10%. After artificial weathering and before investigation in the cone calorimeter, samples were conditioned at 20 °C and 65% relative humidity for two weeks. Profile segments were also subjected to natural weathering for one year according to EN 15534-1 (2018) and EN 927-3. Test specimens were placed in racks that were tilted to an angle of 45 degrees from the horizontal and directed towards the equator. Changes in color were determined as described previously [63]).

### 3.10. SEM-EDX Measurements

Energy dispersive X-ray (EDX) measurements were conducted on an energy dispersive X-ray spectrometer (Oxford Instruments, type X-Max SDD 80 mm^2^) equipped with an SEM (Zeiss, type Leo 1530). The samples were analyzed after plating with a thin carbon layer to render the surface electrically conductive. Electron energy was 5 keV. Samples for measurements were taken on three different areas of each sample with 1–2 mm length.

### 3.11. Mechanical and Physical Properties

Flexural strength and modulus of elasticity of the profiles were determined according to DIN EN 15534-1 (2018) using a three-point-bending test with a material testing machine (BZ1-MM14740.ZW01, Zwick GmbH and Co. KG, Ulm, Germany) and software (TestXpert II). Support span and test specimen length were 500 mm and 600 mm, respectively. Swelling and water uptake were determined with a cold-water submersion test (EN 15534-1). The swelling and weight gain of the profiles were determined according to modified EN 317. Measurement of the dimensions in terms of thickness, width, and length of the specimens was carried out 28 days after the total immersion into water at a temperature of (20 ± 2) °C. The water absorption of the profiles was calculated by differential weighing of the test specimens. The dimensions of the test specimens were 100 mm length and the actual width and thickness of the profiles. For each type of profile, five specimens were tested. Requirements for façade profiles are provided in EN 15534-5. Regarding flexural properties, the only requirement is that deflection under a load of 250 N shall not be higher than 5.0 mm (arithmetic mean value). The span in use shall be declared by the manufacturer. Four specimens per side shall be tested. Further requirements are pertaining to, for example, falling mass impact resistance, durability against biological agents, and moisture resistance under cyclic test conditions but were not tested during the present study.

## 4. Conclusions

Cone calorimeter measurements showed that due to inclusion of APP as fire-retardant in HDPE-wheat straw-composites, the pHRR and THR_600s_ were reduced by up to 64% and 67%, respectively. After 28 days of artificial weathering in a xenon device, the pHRR was not significantly changed and the increase in THR was less than 20% in most formulations. However, because of the relatively high values for the pHRR even before weathering, at best, class D according to EN 13501-1 could be reached based on evaluation according to ETAG 028. The influence of weathering on the fire behavior of the composites can be considered small compared to the difference between fire-retarded and non-fire-retarded composites. Color change of composites with APP was enhanced after artificial and natural weathering compared to composites without fire-retardant.

The best result in terms of the RHR_30save_ after weathering was obtained for co-extruded, solid profiles with FR CROS 490 (242 kW/m^2^). This value was relatively close to the threshold for class C (220 kW/m^2^). Considering that the increase in THR_600s_ was also very low (+2%), it can be concluded that class C could potentially be reached following some optimization.

Some differences in reaction-to-fire performance could be detected between co-extruded solid and co-extruded hollow-core profiles as well as between co-extruded and bulk-protected profiles. However, for all of the profiles tested, in the best case, class D could be reached.

Under the conditions of this study and based on cone calorimeter results and color stability after weathering, FR CROS 490, a APP phase II coated with melamine and formaldehyde-free, performed slightly better compared to Budit 669, a coated intumescent combination based on APP.

Calcium carbonate (CaCO_3_) was included in the tests to serve as placeholder for the fire-retardant. The results indicate that CaCO_3_ contributes to decrease the pHRR in the composites, albeit to a much lower degree than APP. Since CaCO_3_ is non-flammable, it dilutes the total amount of fuel to be consumed.

Neither for co-extruded nor for bulk-protected profiles was any significant leaching of the fire-retardants was detected under the conditions of this study using SEM-EDX measurements.

Regarding flexural strength properties, a requirement that according to DIN EN 15534-5 exists only for elongation at 250 N (<5.0 mm for the arithmetic mean value), was fulfilled by the profiles including the FR. However, long-term studies including creep tests are required to fully evaluate the influence of FR as well as of the wheat straw filler on mechanical performance and durability of the composites. Values for thermal conductivity showed that the profiles are suitable for application as façade materials.

Results from the present study indicate that swelling and water absorption of profiles including FR and wheat straw can be high and need to be carefully monitored as both APP and wheat straw are hygroscopic.

The long-term durability performance of fire-retarded thermoplastic profiles including high amounts of lignocellulose fillers should be further investigated. This should include single-burning-item (SBI) tests with extruded profiles for classification of the materials according to EN 13501-1 before and after weathering.

## Figures and Tables

**Table 1:** Results of UL-94 tests with materials for co-extruded layer. Thickness of pressed sheets was 0.8 mm. Amounts represent wt.%. In each formulation, 3% of coupling agent (MAPE) was included.

Wheat Straw	HDPE	Fire-Retardant	UL-94
70	27	0	Not passed
20	27	50 Magnifin H5-MV	Not passed
30	27	40 Magnifin H5-MV	Not passed
30	27	40 Exolit AP 422	V0
40	27	30 Exolit AP 422	V0
40	27	20 Exolit AP 422; 10 GHL PX 95 HT	V1
40	27	20 Exolit AP 422; 10 Masteret 10460 B2XF	V0
40	27	30 Exolit AP 462	V0
40	27	20 Exolit AP 462; 10 GHL PX 95 HT	V0
40	27	20 Exolit AP 462; 10 Masteret 10460 B2XF	V0
40	27	30 Budit 669	V0
40	27	30 FR CROS 490	V0
50	27	20 Budit 669	Not passed
50	27	20 FR CROS 490	V0

**Table 2:** Results for oxygen index measurements and UL-94 tests. For UL-94 tests, profile segments in 13 mm width (100 mm profile length) were used.

Test	Budit 669	FR CROS 490	Without Fire-Retardant
Limiting oxygen index (LOI)	32	34	20
UL-94 classification	V0	V0	V3

**Table 3:** Fire properties of hollow core (H) and solid (S) profiles before and after artificial weathering (28 days) obtained using a cone calorimeter with 50 kW/m^2^ irradiation. Both types of profiles were co-extruded, with the FR present in the co-ex-layer only. For composition of formulations, see Material and Methods. Values represent the average of three samples with standard deviation in parentheses.

Material	Time to Ignition (s)	pHRR (kW/m^2^)	THR_600s_ (MJ/m^2^)	Mass Loss (wt.%)
Ref H after weathering	21 (0.4)	542 (23.4)	168 (5.4)	33 (0.5)
Ref H before weathering	21 (1.0)	556 (20.1)	186 (4.1)	36 (0.6)
Ref S after weathering	25 (1.5)	498 (14.9)	169 (3.6)	14 (0.3)
Ref S before weathering	24 (0.8)	493 (12.5)	163 (4.9)	15 (0.5)
Bu H after weathering	35 (0.8)	343 (17.6)	137 (3.1)	20 (1.1)
Bu H before weathering	39 (1.3)	320 (11.5)	112 (6.5)	19 (0.8)
Bu S after weathering	37 (1.5)	269 (16.3)	99 (2.7)	10 (0.2)
Bu S before weathering	43 (0.4)	252 (9.8)	95 (4.9)	10 (0.6)
Cr H after weathering	38 (2.0)	307 (10.6)	135 (3.6)	25 (0.5)
Cr H before weathering	40 (1.0)	298 (13.5)	118 (6.2)	25 (0.3)
Cr V after weathering	41 (0.8)	270 (15.6)	100 (4.3)	12 (1.0)
Cr V before weathering	45 (0.7)	257 (13.2)	99 (2.7)	11 (0.8)

**Table 4:** Requirements for fire performance in durability according to ETAG 028 (2012). Heat flux: 50 kW/m^2^.

Building Products Excluding Flooring	RHR_30save_	THR_600s_
Class B products	≤150 kW/m^2^	Increase < 20% compared to before weathering
Class C products	≤220 kW/m^2^	Increase < 20% compared to before weathering

**Table 5:** Results for pHRR, RHR_30save_ and for the increase of the total heat release (THR_600s_) after artificial weathering for 28 days. Classification of the results for hollow-chamber (H) and solid profiles (S) according to ETAG 028 (2012).

Material	pHRR [kW/m^2^]	RHR_30save_ during 600s after T_start_	∆THR_600s_	Classification
Bu H (Budit 669)	343	327	+22%	Neither C nor B
Cr H (FR CROS 490)	307	298	+14%	Neither C nor B
Bu S (Budit 669)	269	250	+4%	Neither C nor B
Cr S (FR CROS 490)	270	242	+2%	Neither C nor B

**Table 6:** Color (∆E*) change of wheat straw-based, fire-retarded, co-extruded profiles after artificial and natural weathering according to DIN EN ISO 4892-2 (cycle 2) and EN 927-3. H: hollow core; S: solid. For composition of the formulations, see Materials and Methods.

Material	Fire-Retardant	∆E* after 300 h Art. Weathering	∆E* after 28 Days Art. Weathering	∆E* after 6 Months Nat. Weathering	∆E* after 12 Months Nat. Weathering
Ref H	Without FR	3.22	6.01	4.90	5.70
Bu H	Budit 669	9.24	11.01	10.80	12.11
Cr H	FR CROS 490	7.67	8.50	9.31	9.02
Ref S	Without FR	4.03	5.02	4.61	4.89
Bu S	Budit 669	6.80	13.47	8.27	8.92
Cr S	FR CROS 490	5.90	8.00	6.97	7.36

**Table 7:** Requirements for swelling and water uptake (DIN EN 15534-5).

Requirement	Threshold Values
Mean values of swelling	≤10% in thickness; ≤1.5% in width; ≤0.6% in length
Individual values of swelling	≤12% in thickness; ≤2.0% in width; ≤1.2% in length
Mean value of water absorption	≤8% in weight
Individual values of water absorption	≤10% in weight

**Table 8:** Thermal conductivity of co-extruded profiles with fire-retardant FR CROS 490. Values represent average of ten measurements; standard deviation in parentheses.

Material	λ [W/m∙K]	U-Value [W/m^2^∙K]
Hollow chamber profile (Cr H)	0.13 (8.6 × 10^−5^)	5.4 (3.5 × 10^−4^)
Solid profile (Cr S)	0.21 (4.1 × 10^−5^)	7.8 (1.5 × 10^−4^)

**Table 9:** TGA results for compounds used in co-extrusion. Abbreviations: T_0_: first onset; T_max_: maximum degradation temperature (maximum rate of degradation); T_f_: final degradation temperature. Values for T_0_, T_max,_ and T_f_ in °C.

Material	T_0_ Step 1	T_max_ Step 1	T_0_ Step 2	T_max_ Step 2	T_0_ Step 3	T_max_ Step 3	T_f_	Mass Loss Step 1 (%)	Mass Loss Step 2 (%)	Mass Loss Step 3 (%)	Residue (%)
Ref	217	270	388	470	-	-	-	30.17	65.09	-	4.72
Bu	216	328	429	482	547	675	827	27.14	32.35	27.23	12.92
Cr	193	320	403	482	529	665	802	19.21	29.02	39.75	9.68

**Table 10:** Chemical composition of straw and wood (percent dry matter). Values for wheat and rice straw from Mo et al. [1] and for wood from Fengel and Wegener [51].

Lignocellulosic Material	Cellulose	Hemicellulose	Lignin	Silica
Wheat straw	39	36	10	6
Rice straw	33	26	7	13
Softwood	43–46	27–30	27	Up to 0.001
Hardwood	43–46	29–37	20–25	Up to 0.001

**Table 11:** TGA results for wheat straw and wood particles. Values represent the average of three measurements (standard deviation in parentheses).

	Mass Loss (wt.%) and Degradation Temperature (°C)			
Material	Step 1	Step 2	Step 3	Residue (wt.%)
Wood flour (C320)	31.93 (1.23); 357	39.39 (1.0); 596	8.90 (0.68); 829	3.68 (2.60)
Wheat straw flour	53.06 (0.63); 356	14.55 (0.21); 427	7.27 (0.29); 591	15.59 (0.73)

**Table 12:** Fire properties of solid profiles before and after artificial weathering (28 days) obtained using a cone calorimeter with 50 kW/m^2^ irradiation. The fire-retardant or CaCO_3_ were present either in the co-extruded layer only or in the bulk of the profile. For composition of formulations, see Material and Methods. Values represent the average of two samples with standard deviation in parentheses.

Material	Time to Ignition (s)	pHRR (kW/m^2^)	THR_600s_ (MJ/m^2^)	Mass loss_600s_ (wt.%)
Co-ex-0 after weathering	18.5 (0.7)	647 (9.1)	195 (1.0)	19 (3.0)
Co-ex-0 before weathering	18.0 (1.4)	639 (5.7)	202 (8.5)	16 (0.8)
Co-ex-FR after weathering	46.0 (4.2)	273 (12.5)	82 (3.2)	8 (0.2)
Co-ex-FR before weathering	46.5 (0.7)	231 (9.2)	66 (4.5)	6 (0.6)
Co-ex-Ca after weathering	35.5 (0.7)	387 (1.5)	97(1.1)	11 (0.7)
Co-ex-Ca before weathering	31.0 (1.4)	384 (6.2)	93 (1.6)	7 (0.3)
Bulk-0 after weathering	23.5 (0.7)	617 (30.7)	257 (8.1)	16 (0.9)
Bulk-0 before weathering	23.0 (2.8)	544 (32.5)	248 (2.6)	14 (0.04)
Bulk-FR after weathering	33.0 (1.4)	301 (9.5)	107 (5.9)	7 (0.6)
Bulk-FR before weathering	75.5 (19.0)	268 (26.9)	100 (4.3)	12 (8.3)
Bulk-Ca after weathering	36.0 (1.4)	412 (28.8)	117 (2.5)	8 (6.7)
Bulk-Ca before weathering	51.5 (13.4)	392 (13.5)	124 (1.5)	6 (1.3)

**Table 13:** Results for RHR_30save_ and for the increase of the total heat release (THR_600s_) for solid profiles after weathering for 28 days. Classification of the results according to ETAG 028 (2012). 0: without any filler; FR: with FR CROS 490; Ca: with calcium carbonate.

Material	pHRR (kW/m^2^)	RHR_30save_ during 600s after T_start_	∆THR_600s_	Classification
Co-ex-0	647	625	−3%	Neither C nor B
Co-ex-FR	273	241	+25%	Neither C nor B
Co-ex-Ca	387	342	+4%	Neither C nor B
Bulk-0	617	596	+4%	Neither C nor B
Bulk-FR	301	283	+7%	Neither C nor B
Bulk-Ca	412	361	−6%	Neither C nor B

**Table 14:** Lightness (∆L*), color (∆a*, ∆b*, and ∆E*), and gloss (∆G) changes of wheat straw-based, fire-retarded, co-extruded profiles after artificial weathering for 28 days according to DIN EN ISO 4892-2 (cycle 2). For composition of the formulations, see Material and Methods. Formulations did not contain any UV absorber, HALS, or acid scavenger.

Material	∆E*	∆L*	∆a*	∆b*	∆G
Co-ex-0	7.36	7.33	0.54	0.39	0.1
Co-ex-FR	12.33	11.72	3.15	2.15	−0.4
Co-ex-Ca	10.80	10.23	2.67	2.20	−6.2
Bulk-0	3.41	3.25	0.87	0.55	1.8
Bulk-FR	30.07	30.00	1.27	1.65	−13.0
Bulk-Ca	13.29	12.74	3.16	2.04	−1.7

**Table 15:** Results of elemental analysis in atomic-% for profiles with FR CROS 490 before and after 28 days artificial weathering. Co-ex: co-extruded profiles; bulk: solid profiles (uncapped). Standard deviation in parentheses.

Material	C	N	O	Si	P	Fe
Co-ex-FR before w.	89.7 (0.8)	2.1 (0.1)	6.6 (0.4)	0.47 (0.28)	0.8 (0.1)	0.16 (0.01)
Co-ex-FR after w.	85.0 (0.4)	3.1 (0.1)	9.9 (0.3)	0.16 (0.06)	1.6 (0.1)	0.20 (0.02)
Bulk-FR before w.	85.0 (0.3)	3.2 (0.1)	9.9 (0.2)	0.15 (0.02)	1.6 (0.1)	0.21 (0.01)
Bulk-FR after w.	85.1 (3.7)	2.2 (0.7)	10.3 (2.4)	0.34 (0.03)	1.6 (0.6)	0.30 (0.03)

**Table 16:** Series 1: Composition of formulations for comparison of hollow core (H) and solid (S) profiles. Both types of profiles were co-extruded, with the FR present in the co-extruded layer only. Percentages represent weight-%. In each formulation, 3% of coupling agent (MAPE) was used. In addition, co-extruded formulations contained 4% lubricant; 2.75% pigment masterbatch; 0.25% Irganox B225; 0.1% Tinuvin 326; 0.4% Tinuvin 783; and 0.15% Hycite 713.

Material	HDPE (56020S, Total)	Wheat Straw	Lubricant	Fire-Retardant
Ref H (co-ex-layer)	65.9	24.1	4.0	0
Ref S (co-ex-layer)	65.9	24.1	4.0	0
Bu H (co-ex-layer)	25.9	24.1	4.0	40.0 Budit 669
Bu S (co-ex-layer)	25.9	24.1	4.0	40.0 Budit 669
Cr H (co-ex-layer)	25.9	24.1	4.0	40.0 FR CROS 490
Cr S (co-ex-layer)	25.9	24.1	4.0	40.0 FR CROS 490
Core	46.0	50.0	1.0	0

**Table 17:** Series 2: Formulations for comparison of profiles with FR in the co-extruded layer only or with FR in the bulk. In each case, solid profiles were extruded. Amounts represent wt.%. Each formulation (exception: core for profiles with co-extruded layers) contained 3% MAPE, 4% lubricant, 2.75% color masterbatch, and 0.25% stabilizer (Irganox B225). The core layer for the co-extruded profiles contained only 3% MAPE and 1% lubricant.

Material	Description	HDPE (56020S, Total)	HDPE (5502, Total)	Wheat Straw	FR CROS 490	Calcium Carbonate
Co-ex-0	Co-ex-layer	0	65.9	24.1	0	0
Co-ex-FR	Co-ex-layer	0	25.9	24.1	40	0
Co-ex-Ca	Co-ex-layer	0	25.9	24.1	0	40
Core	Core for co-ex	46	0	50.0	0	0
Bulk-0	Bulk protected	65.9	0	24.1	0	0
Bulk-FR	Bulk protected	25.9	0	24.1	40	0
Bulk-Ca	Bulk protected	25.9	0	24.1	0	40

## Data Availability

Not applicable.

## Sample Availability

Samples of the compounds are available from the authors.
